# Ultrasound initiated tumor catalytic PANoptosis by mesoporous piezoelectric nanocatalysts

**DOI:** 10.1186/s40779-025-00629-9

**Published:** 2025-07-30

**Authors:** Xuan-Shou Xu, Wei-Wei Ren, Heng Zhang, Dong-Liang Huo, Qi Lyu, Mei-Xiao Zhan, Hui-Xiong Xu, Li-Ying Wang, Min-Feng Huo, Jian-Lin Shi

**Affiliations:** 1 Department of Medical Ultrasound, Shanghai Tenth People’s Hospital, Shanghai Frontiers Science Center of Nanocatalytic Medicine, the Institute for Biomedical Engineering and Nano Science School of Medicine, Tongji University School of Medicine, Shanghai, 200072 China; 2 Department of Ultrasound, Zhuhai People’s Hospital (the Affiliated Hospital of Beijing Institute of Technology, Zhuhai Clinical Medical College of Jinan University), Zhuhai, 519000 Guangdong China; 3 Department of Ultrasound, Zhongshan Hospital, Institute of Ultrasound in Medicine and Engineering, Fudan University, Shanghai, 200032 China; 4 Shanghai Institute of Ceramics, Chinese Academy of Sciences, Research Unit of Nanocatalytic Medicine in Specific Therapy for Serious Disease, Chinese Academy of Medicine Science (2021RU012), Shanghai, 200050 China

**Keywords:** Piezoelectric nanocatalyst, Tumor PANoptosis, Sonodynamic therapy, Immunotherapy, Mesopore engineering

## Abstract

**Background:**

PANoptosis has been identified as a robust inflammatory cell death pathway triggered upon host defense against invaded pathogens such as bacteria and viruses, however, pathogen-free tumor PANoptosis has not been achieved yet. Reactive oxygen and nitrogen species capable of inducing robust and diverse cell death pathways such as pyroptosis, apoptosis, and necroptosis are supposed to be the potential triggers for tumor PANoptosis by ultrasound (US)-controlled sono-piezodynamic therapy.

**Methods:**

S-nitrosothiols (SNO)-zinc peroxide (ZnO_2_)@cyclic dinucleotide (CDN)@mesoporous tetragonal barium titanate (mtBTO) nanoparticles (NZCB NPs) were synthesized by hydrothermal method with subsequent annealing, in situ growth, and finally surface functionalization. Scanning electron microscopy, transmission electron microscopy, X-ray diffraction, atomic force microscopy, Fourier transform infrared spectroscopy, and electron spin resonance were used for materials characterizations. Murine melanoma B16 cells are employed to investigate the in vitro US-initiated tumor PANoptosis by NZCB NPs. In vivo US-initiated tumor PANoptosis was investigated on B16 tumor-bearing C57BL/6J mice.

**Results:**

A “boiling-bubbling” strategy is developed to endow the piezoelectric BTO nanocatalysts, with mesoporous architecture, which enables the encapsulation of the immune-agonist CDN (9.4 wt%) to initiate innate immunity of the host. Then, SNO-functionalized ZnO_2_ was further employed to cap the mesoporous nanocatalysts, forming multifunctional piezocatalytic NZCB NPs. Under US irradiation, intracellular massive reactive oxygen and nitrogen species such as superoxide anion radicals, nitric oxide (NO), and peroxynitrite (ONOO^−^) could be produced from the piezoelectric NZCB NPs, which, synergized with CDN-triggered antitumoral immunity, lead to highly immunogenic tumor PANoptosis by NZCB NPs through the tumor microenvironment remodeling. Intratumoral injection of NZCB NPs leads to substantial tumor PANoptosis with immune potentiation, ultimately destroying the tumor xenografts effectively.

**Conclusion:**

The present work presents the mesostructure design of piezocatalytic nanomaterials and the crosstalk between oxidative stress and antitumor immunity within the tumor, facilitating promising tumor PANoptosis by nanocatalytic oxidation with high effectiveness and biocompatibility.

**Supplementary Information:**

The online version contains supplementary material available at 10.1186/s40779-025-00629-9.

## Background

Piezoelectric nanomaterials have attracted extensive research attention across various medical applications, encompassing drug delivery [1, 2], bone and cartilage regeneration [3–6], wound healing [7], neuron stimulation [8, 9], and tumor therapy [10, 11]. With a piezoelectrically active space group, the positively and negatively charged centers will be separated upon external pressure, altering the surface charge profile of the piezoelectric nanomaterials [12]. A notable advancement in the application of piezoelectric nanomaterials is the emergence of piezoelectric catalytic nanomedicine, initiated by the irradiation of medical ultrasound (US) for sono-piezodynamic therapy (SPDT) [11, 13–15]. Such a therapeutic approach holds considerable promise for achieving localized US-controlled tumor ablation [16–20]. SPDT also triggers the separation of reactive electrons and holes for respective piezoelectric catalysis, resulting in the generation of reactive oxygen species (ROS) such as hydroxyl radical (·OH) and superoxide anion radicals (·O_2_^−^) [21, 22]. Several piezoelectric nanomaterials have been developed for biomedical applications [10, 23, 24]. For instance, barium titanate (BTO), a prototypical piezoelectric material, has been used for US-activated SPDT due to its high biocompatibility and robust piezoelectricity [11, 21, 25, 26]. Nevertheless, the performance of these piezoelectric nanocatalysts-enabled SPDT was restricted by tumor hypoxia, limiting the catalytic transformation from oxygen (O_2_) molecules to cytotoxic ·O_2_^−^. Furthermore, to obtain robust anti-tumor performance, prominent anti-tumor immunity is hopefully synergized. Mesopore engineering as well as surface functionalization on BTO is specifically challenging yet appealing to introduce synergistic therapeutic strategies to conquer the limitation of SPDT enabled by bare BTO [27], ultimately boosting the therapeutic outcome against malignant tumors.

In addition, SPDT is capable of inducing immunogenic cell death against malignant tumors. During SPDT, tumor-associated antigens and damage-associated molecular patterns can be generated from dying tumor cells to mediate the maturation of dendritic cells, antigen presentation, and migration to tumor-draining lymphoid nodes for T cell activation [28, 29]. Nevertheless, such stimulation is too weak to induce prominent and sustained anti-tumor immunities since most of the immunogenic damage-associated molecular patterns were degraded inside the apoptotic body along with tumor apoptosis. Highly immunogenic cell death patterns are demanding to achieve the goal of tumor destruction by piezoelectric nanocatalysts-enabled SPDT.

PANoptosis, a form of robust inflammatory programmed cell death pathway regulated by the PANoptosome has been identified during host defense against pathogens such as bacteria and viruses. Infected cells can responsively assemble the PANoptosome to trigger inflammatory PANoptosis and host immunity, exhibiting pivotal immunogenic features including pyroptosis, apoptosis, and necroptosis. PANoptosis is believed to potentiate the anti-tumor immunity substantially, benefiting the tumor therapeutics with high effectiveness.

To guarantee biocompatibility and biosafety, potential pathogen-free strategies to initiate tumor PANoptosis are of substantial significance, yet currently unknown. Reactive oxygen and nitrogen species are potent intracellular messengers that could induce robust and diverse cell death pathways, such as pyroptosis, apoptosis, and necroptosis. These species are potential triggers for tumor PANoptosis during SPDT. Therefore, we are intensively inspired to construct a pathogen-free nanocatalytic oxidative strategy inside functionalized BTO to initiate SPDT to potentiate nanocatalytic tumor PANoptosis for robust tumor therapeutics. Melanoma is a highly malignant superficial skin cancer with a median survival period of less than 1-year and a 5-year survival rate below 10% [30]. Due to its typical resistance to apoptosis, effective strategies to induce non-apoptotic programmed cell death against the tumor are highly appealing [31]. Therefore, this study will focus particularly on such tumor cells.

## Methods

### Synthesis of mesoporous tetragonal BTO (mtBTO) nanoparticles (NPs) with different pore sizes

Primitive tetragonal BTO (tBTO) NPs were synthesized according to the previous report [11]. mtBTO NPs were obtained via a “boiling-bubbling” strategy. Specifically, 0.1 g of tBTO NPs were introduced into a flask containing 70 ml hydrogen peroxide (H_2_O_2_) solution (w/w, 30%) with ultrasonication for 25 min. The resulting mixture was transferred to a 100 ml high borosilicate bottle, and placed in an oil bath for hydrothermal reaction under temperatures of 125 or 135 °C for either 2 or 4 h. The final product was centrifuged and collected, denoted as mtBTO NPs.

### Synthesis of ZnO_2_@mtBTO NPs

A total of 27 mg ZnCl_2_ was dispersed in 3 ml ddH_2_O to form solution A, and 10 mg bovine serum albumin (BSA) powder was dispersed in 1 ml ddH_2_O to form solution B. Solution A and B were added dropwisely into the solution containing mtBTO NPs (25 mg/ml, 1 ml) with stirring. Then, 3 ml H_2_O_2_ solution (w/w, 30%) was added dropwisely and stirred for 15 min. 1 ml sodium hydroxide (NaOH) solution (2 mol/L) was then added dropwisely and reacted for 4 h. As the reaction proceeded, the color of the solution turned milky. After stirring for another 10 h at room temperature, ZnO_2_@mtBTO NPs were obtained after centrifugation and washed with ddH_2_O twice.

To encapsulate cyclic dinucleotide (CDN), 2′,3′-cyclic GMP-AMP synthase (cGAMP) sodium (0.5 mg) was dispersed in the solution containing mtBTO NPs (25 mg/ml, 1 ml) and stirred at 4 °C for 2 h. Afterward, H_2_O_2_ and NaOH solutions were added as described above to obtain CDN-loaded ZnO_2_@mtBTO NPs, denoted as ZnO_2_@CDN@mtBTO NPs.

### Synthesis of S-nitrosothiols (SNO)-ZnO_2_@mtBTO NPs and NZCB NPs

ZnO_2_@mtBTO NPs were first linked with –SH for further SNO transformation. ZnO_2_@mtBTO NPs (25 mg) and 10 mg BSA were added to 5 ml phosphate buffer saline (PBS) containing 5 mmol/L EDTA. Then, Traut’s reagent (1 mg) was added to the solution and stirred at 4 °C for 2 h. The solution was then centrifuged at 5000 rpm for 3 min at 4 °C and washed twice with deionized water to obtain ZnO_2_@mtBTO-SH NPs. Afterward, CH_3_OH and CH_2_Cl_2_ were mixed in V/V = 5 and stirred at 4 °C in the dark. Tert-butyl nitrite (1 ml) was then added dropwisely and stirred for 5 min. BSA (20 mg) and ZnO_2_@mtBTO-SH NPs (25 mg) were then added and reacted for further 4 h under 4 °C in the dark. Besides, to obtain NZCB NPs, CDN-loaded ZnO_2_@mtBTO NPs were used for –SH linkage and subsequent thiol transformation.

### Measurement of H_2_O_2_ and O_2_ released from SNO-ZnO_2_@mtBTO NPs

A H_2_O_2_ assay kit was used to detect the production of H_2_O_2_ from SNO-ZnO_2_@mtBTO NPs at varied concentrations (15.6, 31.3, 62.5, 125.0, 250.0, and 500.0 μg/ml) under pH 7.4 and 6.0 according to the protocols. O_2_ concentration from SNO-ZnO_2_@mtBTO NPs at 500 μg/ml under pH 7.4, 6.5 and 5.0 was recorded by oxygen electrode.

### Measurement of NO release from SNO-ZnO_2_@mtBTO NPs

Nitric oxide (NO) release from SNO-ZnO_2_@mtBTO NPs was quantitatively measured using NO assay kit based on the Griess method according to protocols. SNO-ZnO_2_@mtBTO NPs at varied concentrations of 7.81, 15.63, 31.25, 62.50, and 125.00 μg/ml were detected with NO production with or without US irradiation (1.0 W/cm^2^, 1 min).

### Methylene blue (MB) degradation evaluation

The mtBTO NPs, ZnO_2_@mtBTO NPs, and SNO-ZnO_2_@mtBTO NPs (200 μg/ml, 2 ml) were firstly mixed with MB solution (0.2 mmol/L, 0.2 ml) and stirred overnight in the dark to diminish the absorption disturbance. Afterwards, the assay solution was treated with US (1.0 W/cm^2^, 50% duty ratio) for varied periods (1, 3, 5, and 10 min), followed by centrifugation and supernatant collection for ultraviolet–visible (UV–vis) spectroscopy measurements from 400 to 800 nm.

### Singlet oxygen (^1^O_2_) and ·OH evaluations

1,3-Diphenylisobenzofuran (DPBF) probe was applied as the trapping agent for ^1^O_2_. Typically, mtBTO NPs, ZnO_2_@mtBTO NPs, and SNO-ZnO_2_@mtBTO NPs (200 μg/ml, 500 μl) were added into DPBF solution (0.5 mg/ml, 1.5 ml), followed by US irradiation at 1.0 W/cm^2^ for varied periods (1, 3, 5, and 10 min). Then the mixed solution was detected with optical absorbance from 280 to 480 nm in a UV–vis spectrometer. For electron spin resonance (ESR) experiments, solution containing DMPO (50 mmol/L, 100 μl) with mtBTO NPs, ZnO_2_@mtBTO NPs, or SNO-ZnO_2_@mtBTO NPs (20 μg/ml, 900 μl) were transferred to quartz capillaries for ESR detection after US irradiation (1.0 W/cm^2^, 1 min, 50% duty ratio).

### Cell culture and endocytosis assays of fluorescein isothiocyanate (FITC)-labeled mtBTO NPs

Murine melanoma B16 tumor cells (Cat#: TCM36) were purchased from the Cell Bank, the Committee of Type Culture Collection of the Chinese Academy of Sciences. These cells have not been listed in the cross-contaminated or misidentified cell lines (version 8.0, 2016) and are qualified based on cell line tests, including morphology identification, isoenzymes, and mycoplasma.

mtBTO NPs were labeled with FITC via stirring for cellular endocytosis assay. Murine melanoma B16 tumor cells were incubated with 1640 medium containing 10% fetal bovine serum and 1% penicillin–streptomycin under 37 °C in a humidified incubator with 5% CO_2_. B16 cells were inoculated at a density of 1 × 10^5^ into confocal laser scanning microscopy (CLSM)-specific dishes and incubated at 37 °C for 24 h. The medium was then replaced with FITC-mtBTO NPs containing fresh medium (50 μg/ml) and incubated for 1, 2, 3, and 4 h. At the end of incubation, the medium was removed and the cells were washed 3 times with PBS, followed by staining with 4′,6-diamidino-2-phenylindole (DAPI) for 15 min. CLSM imaging experiments were performed on an Olympus FV1000 laser scanning microscope.

### Quantitative reverse transcription polymerase chain reaction (RT-qPCR)

The RNA of B16 tumor cells was extracted using TRIzol and the RNA was converted to cDNA sequences via reverse transcription using PrimerScript RT reagent kit (TaKaRa, Tokyo, Japan). A STBR RT-PCR kit (Vazyme, q712) was used for RT-qPCR analysis. Primers used in this manuscript were listed in Additional file 1: Table S1.

### Flow cytometric identification of macrophages and dendritic cells

RAW264.7 cells were purchased from Cell Bank of the Chinese Academy of Sciences and were inoculated at 2 × 10^6^ cells/well in 6-well plates and incubated with ZnO_2_@mtBTO NPs, SNO-ZnO_2_@mtBTO NPs, and NZCB at 50 μg/ml for 4 h in 1640 medium with pH = 6.5, followed by US treatments (1.0 W/cm^2^, 1 min, 50% duty ratio) and further incubation for 2 h. At the end of treatments, RAW264.7 cells were collected and stained with CD206-PE and CD86-APC after cell permeabilization for flow cytometry analysis.

Bone marrow dendritic cells (BMDCs) were isolated from the bone marrow of C57BL/6J mice (*n* = 3) and inoculated at 4 × 10^6^ cells/well in the lower chamber of the transwell. B16 cells were pretreated with ZnO_2_@mtBTO NPs, SNO-ZnO_2_@mtBTO NPs, and NZCB with or without US irradiations as previously described. Then, the resultant B16 cells were collected and placed in the upper chamber of the transwell and incubated with BMDCs overnight. Finally, BMDCs were collected and stained with CD11c-APC, CD80-PE and CD86-VF660 for flow cytometry identification.

### In vivo antitumor evaluations

All the animal procedures were performed under the protocol approved by the Laboratory Animal Center of Shanghai Tenth People’s Hospital (SHDSYY-2023-6600), Shanghai, China. For in vivo therapeutics against subcutaneous tumor xenografts, female C57BL/6J mice (4 weeks old, *n* = 30) were purchased from Beijing Vital River Laboratory Animal Tech. Co., Ltd. The mice were randomly divided into 6 groups, including control, US, mtBTO NPs + US, ZnO_2_@mtBTO NPs + US, SNO-ZnO_2_@mtBTO NPs + US, and NZCB NPs + US. All mice were subcutaneously injected with the suspension of B16 cells (100 μl, 10^6^ cells per mouse). After 7 d, the tumor volume of xenografts reached 90 mm^3^ and mice were intratumorally injected with saline, mtBTO, ZnO_2_@mtBTO, SNO-ZnO_2_@mtBTO, and NZCB dispersion (100 μl, 20 mg/kg) accordingly. US irradiation was carried out 4 h post-administration. Afterward, body weights and tumor dimensions were recorded every other day. According to the animal guidelines, mice with xenograft volume over 1000 mm^3^ were sacrificed by painless cervical dislocation.

In another batch of in vivo experiments, all mice were euthanized after 10 d of therapeutic evaluation, followed by blood collection for blood routine and blood biochemical analyses. Besides, major organs including the heart, liver, spleen, lung, and kidneys were collected for pathological hematoxylin and eosin analysis. Tumor tissues were either subjected to paraformaldehyde fixation for pathological analysis, immunofluorescence staining, or preserved in liquid nitrogen for mRNA sequencing and Western blotting analysis.

For immune infiltration evaluation, tumor tissues were collected from mice and treated with 1500 U/ml collagenase (Merck, USA), 1000 U/ml hyaluronidase (Merck, USA), and Sigma DNase (Merck, USA) at 37 °C for 30 min. Cells were filtered through a nylon mesh filter and washed with PBS containing 1% FBS. Single-cell suspensions were stained by incubation with live/dead, anti-CD3, CD4, CD8, CD11b, F4/80, CD86, CD206 antibodies. These cells were then washed with PBS containing 1% FBS and analyzed using flow cytometry.

### Statistical analysis

Data are displayed as the mean ± SD. The data significance is analyzed following the paired Student’s *t-*test and One-Way ANOVA. *P* < 0.05 was considered statistically significant.

## Results

### Synthesis and characterization of mtBTO NPs

**Fig. 1:**
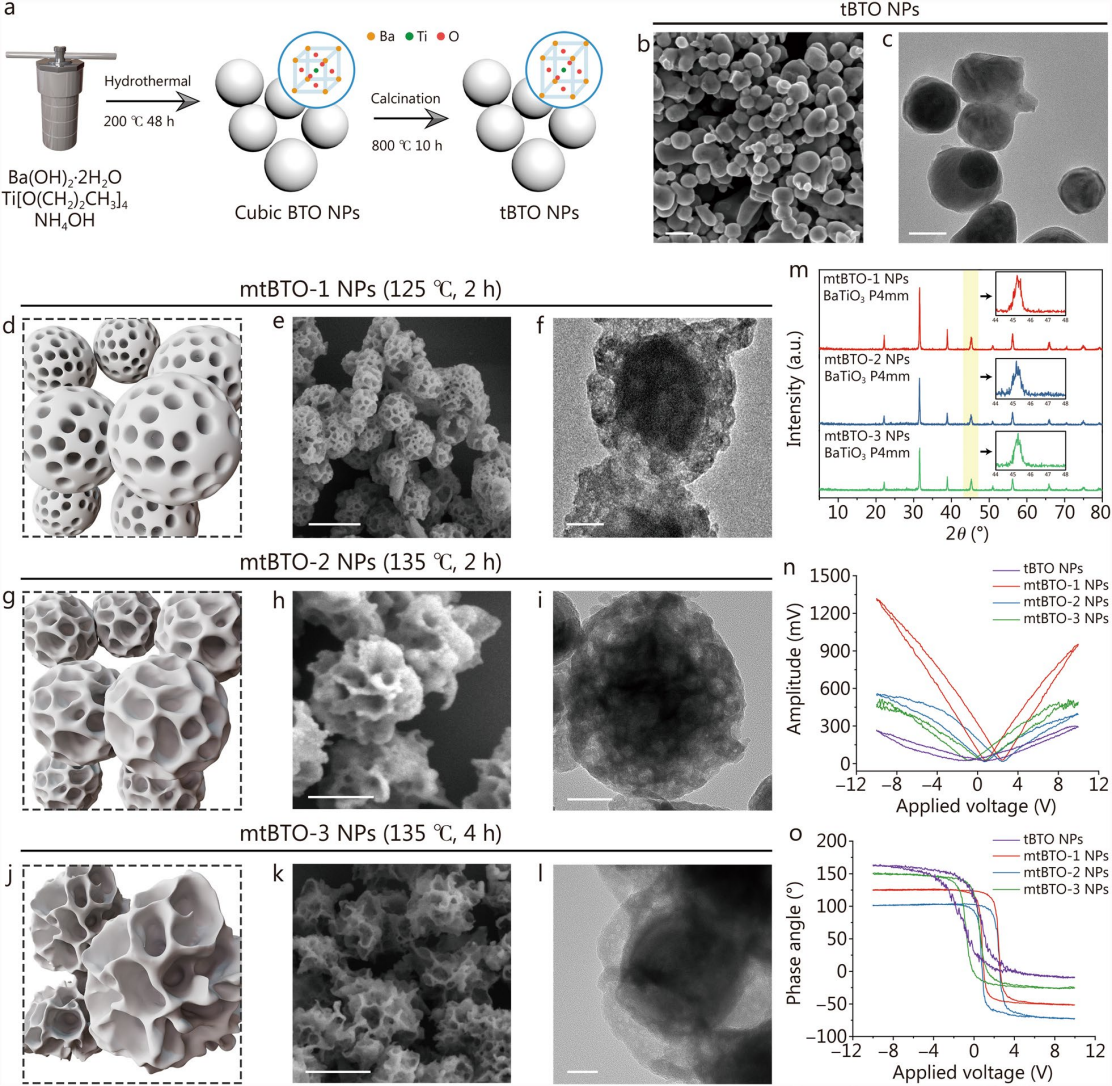
Characterization of mtBTO NPs with controllable pore sizes. **a** Schematic illustration of the preparation of mtBTO NPs. SEM (**b**) and TEM (**c**) images of the morphology of tBTO NPs. Schematic illustration (**d**), SEM (**e**), and TEM (**f**) images of mtBTO-1 NPs. Schematic illustration (**g**), SEM (**h**), and TEM (**i**) images of mtBTO-2 NPs. Schematic illustration (**j**), SEM (**k**), and TEM (**l**) images of mtBTO-3 NPs. **m** XRD spectra of mtBTO-1, mtBTO-2, and mtBTO-3 NPs. Typical amplitude-voltage “Butterfly” curves (**n**) and phase-voltage hysteresis loops from the piezoresponse atomic force microscopy (**o**) of tBTO, mtBTO-1, mtBTO-2, and mtBTO-3 NPs. Scale bar = 200 nm (**b, f, i, l**) and 50 nm (**c, e, h, k**). NPs nanoparticles, SEM scanning electron microscopy, transmission electron microscopy, XRD X-ray diffraction, tBTO tetragonal barium titanate, mtBTO mesoporous tetragonal barium titanate

Primitive BTO NPs were synthesized by the hydrothermal treatment (200 °C, 48 h) of the precursors containing Ba and Ti. These NPs have cubic morphology with an average size of 120.3 nm (Additional file 1: Fig. S1). According to the X-ray diffraction (XRD) pattern, primitive BTO NPs are in cubic syngony with symmetric centers (denoted as cubic BTO NPs, Additional file 1: Fig. S2). Based on crystallography, the piezoelectricity of BTO NPs originates from the piezo-initiated separation of positive charge centers (contributed by Ba, Ti) and negative charge centers (contributed by O). Post-annealing process (calcination under 800 °C for 10 h) enables the transformation from cubic BTO NPs to tBTO NPs (Fig. 1a), as revealed by the characteristic peak splitting at 2*θ* = 45° during XRD measurement (Additional file 1: Fig. S3). tBTO NPs exhibit a dense structure in both scanning electron microscopy (SEM) (Fig. 1b) and transmission electron microscopy (TEM) (Fig. 1c), limiting their biological applications in drug delivery and surface modifications.

To create mesopores in tBTO NPs, we invented the “boiling-bubbling” strategy using a high concentration of H_2_O_2_ for the hydrothermal treatment of tBTO NPs. Specifically, a mixed solution containing H_2_O_2_ and tBTO NPs was transferred into a sealed flask and heated in an oil bath under varied conditions of temperature and time. Upon investigating the resultant products using SEM, we found that H_2_O_2_-based hydrothermal treatment initiated the mesopore-forming etching process of tBTO NPs. Treatment temperature, reaction time, and tBTO NPs/H_2_O_2_ ratio are key factors that affect the formulated dimension of the mesopores. When the etching temperature and reaction time decreased to 110 °C for 2 h and 125 °C for 1 h, the BTO NPs surface was identified as non-mesoporous (Additional file 1: Fig. S4). Along with the increased treatment temperature from 125 to 135 °C and reaction time from 2 to 4 h, the morphology of tBTO NPs gradually changed from non-porous to porous architecture with increasing mesopores dimension (Fig. 1d, g, j). We assumed that under the sealed hydrothermal treatment, boiling H_2_O_2_ solvents led to oxidative etching against tBTO NPs, generating O_2_ bubbles for pore formation.

When the reaction condition was set to 125 °C for 2 h, the resultant mtBTO-1 NPs exhibited a particulate dimension of 211 nm and a mean pore dimension to be 29.95 nm (Fig. 1e). TEM was employed to further confirm the etching process and mesoporous formulation on mtBTO-1 (Fig. 1f). When the reaction condition was set to 135 °C for 2 h, the resultant mtBTO-2 NPs exhibited a flower-like morphology with a mean particulate dimension of 241 nm and a mean pore dimension of 40.83 nm (Fig. 1h). TEM images also identified the increased pore sizes in comparison with mtBTO-2 NPs (Fig. 1i). When the reaction condition was set to 135 °C for 4 h, the resultant mtBTO-3 NPs exhibited a larger particulate dimension of 388.3 nm and a mean pore dimension of 76.49 nm, indicating the overall expansion of the NPs and the formulation of the large pores (Fig. 1k, l). Consistently, the hydrodynamic diameter assayed according to the dynamic light scattering method verified the increasing particle sizes of 197.6 nm, 267.2 nm and 310.7 nm separately for mtBTO NPs with varied pore structures (Additional file 1: Fig. S5). The N_2_ adsorption–desorption isotherms were assayed for the mtBTO NPs treated with different conditions. The specific surface area of mtBTO-1, mtBTO-2, and mtBTO-3 NPs was calculated as 14.46, 23.25 and 28.84 m^2^/g respectively according to Brunauer-Emmett-Teller method. With the Barrett-Joyner-Halenda method, the pore size was assayed to be 18.09, 22.36, and 15.68 nm, respectively. The results are statistically inconsistent with the microscopic observation, possibly due to the presence of non-penetrating pores limiting the adsorption and desorption of N_2_, reducing the surface area and pore diameter assayed from this detection method (Additional file 1: Fig. S6).

As revealed from the XRD patterns, mtBTO NPs preserve identical syngony and phase of tBTO NPs after varied H_2_O_2_ treatments, as revealed by the XRD patterns (Fig. 1m). mtBTO-1 and mtBTO-2 NPs still exhibited characteristic peak splitting at 2*θ* = 45°, identifying tetragonal structure with piezoelectric property for promising SPDT applications. For mtBTO-3 NPs, peak splitting can hardly be observed, possibly due to the structural distortion (Additional file 1: Fig. S7). Atomic force microscopy indicated increased roughness of mtBTO NPs compared with solid tBTO NPs, originating from the surface pore structures (Additional file 1: Fig. S8). To investigate the piezoelectric properties of BTO before and after H_2_O_2_ treatment, we conducted piezo-responsive atomic force microscopic tests for tBTO NPs and mtBTO NPs under bias voltages ranging from − 10 to 10 V. Both tBTO and mtBTO NPs exhibited characteristic butterfly curves (Fig. 1n), revealing the mechanical responses to varied applied voltages. Interestingly, mtBTO-1 NPs exhibited optimal piezoelectric performance, which could be attributed to the intact skeleton structure and increased vacancies after H_2_O_2_ treatments. Moreover, reversing the direct current bias electric field led to changes in phase angles, suggesting that both tBTO and mtBTO maintain good piezoelectric properties (Fig. 1o).

Based on these experiments, the as-obtained mtBTO-1 NPs feature abundant mesopores with adequate diameters of 20–40 nm for effective drug encapsulation and delivery. Therefore, mtBTO-1 NPs treated with H_2_O_2_ under 125 °C for 2 h were employed for further experiments and characterizations. Selected area electron diffraction indicates (001) as the primary direction. Lattice spacing was measured to be 0.20 nm and 0.34 nm on high-angle annular dark-field-scanning transmission electron microscopy images, corresponding to (100) and (001) crystal facets (Additional file 1: Fig. S9). Energy dispersive spectral elemental mapping indicates the surface mesopore distribution and intact distribution of O, Ti and Ba in the core of BTO (Additional file 1: Fig. S10). From the Fourier transform infrared spectra (FTIR), additional hydroxyl groups could be found on the surface of mtBTO NPs after H_2_O_2_ hydrothermal treatment, imparting improved water dispersity (Additional file 1: Fig. S11). X-ray photoelectron spectroscopic spectra show that the Ti2p spectra between tBTO and mtBTO-1 NPs remain unchanged, while the position of the Ba3d peaks of mtBTO-1 NPs shifts to a higher energy level as compared to tBTO NPs, suggesting the presence of potential lattice defects (Additional file 1: Fig. S12). mtBTO-1 NPs have a wide bandgap of 2.94 eV according to the diffuse reflection UV-vis spectrum (Additional file 1: Fig. S13). The electronic potential was measured and calculated to be − 0.86 V (against the Ag/AgCl reference electrode) from the Mott-Schottky test. Therefore, the conduction band edge and valence band edge are calculated to be − 0.66 V (against the reversible hydrogen electrode) and 2.28 V, respectively.

### Construction of NZCB NPs

**Fig. 2:**
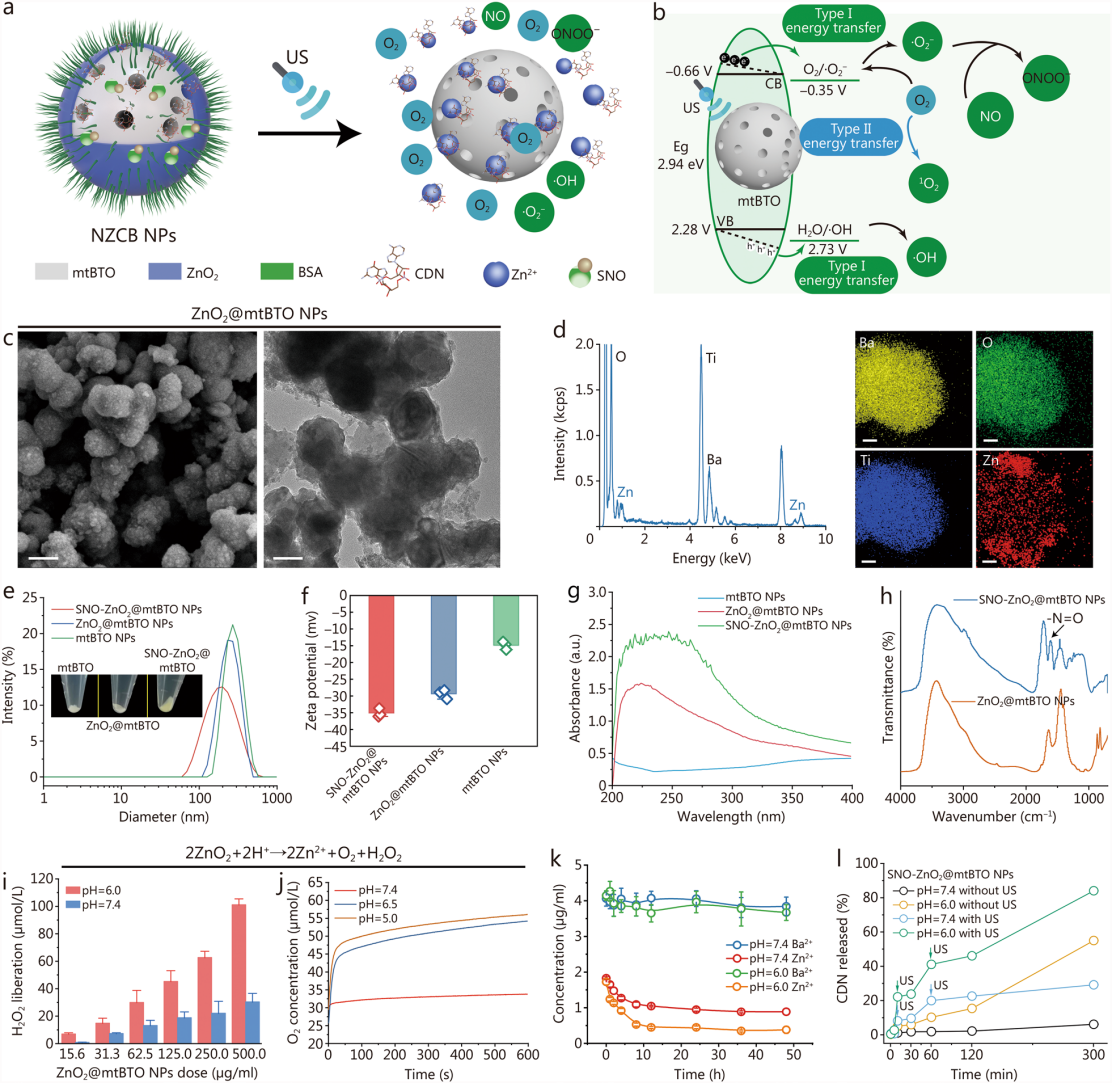
Construction and physicochemical properties of NZCB NPs. **a** Schematic illustration of NZCB NPs and the US-initiated decomposition and drug release. **b** Mechanistic illustration of US-initiated cascade radical production. **c** SEM (scale bar = 100 nm) and TEM images (scale bar = 50 nm) of ZnO_2_@mtBTO NPs. **d** EDS and corresponding elemental mapping of Ba, O, Ti, and Zn of ZnO_2_@mtBTO NPs (scale bar = 25 nm). Hydrodynamic diameter (**e**) and zeta potential (**f**) of mtBTO, ZnO_2_@mtBTO, and SNO-ZnO_2_@mtBTO NPs. **g** UV–vis spectra of mtBTO, ZnO_2_@mtBTO, and SNO-ZnO_2_@mtBTO NPs. **h** FTIR spectra of ZnO_2_@mtBTO and SNO-ZnO_2_@mtBTO NPs. **i** H_2_O_2_ production of ZnO_2_@mtBTO NPs (15.6, 31.3, 62.5, 125.0, 250.0, and 500.0 μg/ml) under pH 6.0 and 7.4. **j** O_2_ production curves of ZnO_2_@mtBTO NPs (500 μg/ml) in 10 min under pH 7.4, 6.5, and 5.0. **k** Time-course elemental concentration of Zn^2+^ and Ba^2+^ of SNO-ZnO_2_@mtBTO NPs at 10 μg/ml after centrifugation under pH 7.4 and 6.0 via ICP. **l** CDN release curves of NZCB NPs under pH 7.4 and 6.0 with or without US treatment. US ultrasound, SEM scanning electron microscopy, TEM transmission electron microscopy, EDS energy dispersive X-ray spectroscopy, UV-vis*-* ultraviolet-visible spectroscopy, FTIR Fourier transform infrared spectroscopy, ICP inductively coupled plasma spectroscopy, ·OH hydroxyl radical, ^1^O_2_ singlet oxygen radical, ·O_2_^−^ superoxide radical, ONOO^*−*^ peroxynitrite, NZCB S-nitrosothiols (SNO)-zinc peroxide (ZnO_2_)@cyclic dinucleotide (CDN)@mesoporous tetragonal barium titanate (mtBTO), NPs nanoparticles, BSA bovine serum albumin, VB valence band, CB conduction band

To potentially initiate the oxidative inflammatory cascade conditions against the tumor, we employed the mtBTO NPs to encapsulate the STING agonist (2′,3′-cGAMP) into the mesopores to activate the innate anti-tumor immunity against the tumor [32]. The encapsulated CDN@mtBTO NPs were subsequently sealed and capped with ZnO_2_ NPs through an in-situ self-assembly method with BSA, H_2_O_2_, and NaOH, forming ZnO_2_@CDN@mtBTO NPs. Additional SNO was further functionalized onto ZnO_2_ NPs, forming NZCB NPs to facilitate the formation of ONOO^−^ via the interaction of NO and ·O_2_^−^ (Fig. 2a). Mechanically, the energy band tilt of mtBTO under US stimulation initiates radical production via H_2_O/·OH and O_2_/·O_2_^−^ reactions. Then, the ZnO_2_ layer exhibits an acid-responsive decomposition to release H_2_O_2_ and O_2_, thereby amplifying the production efficiency of ^1^O_2_ and ·O_2_^−^ from O_2_ by piezoelectric mtBTO NPs when subjected to US. SNO group releases NO molecules under US waves and forms ONOO^−^ radicals with ·O_2_^−^, therefore, realizing US-controlled cascade (ROS and reactive nitrogen species (RNS) generation of NZCB NPs (Fig. 2b).

Successful coating of ZnO_2_ onto mtBTO NPs was validated by the disappearance of the mesoporous architecture, as confirmed by the SEM and TEM images of ZnO_2_@mtBTO NPs (Fig. 2c). The results of energy dispersive X-ray spectroscopy and corresponding elemental mapping (Fig. 2d) indicate the predominant presence of Zn element on the surface, collectively confirming the successful growth of the ZnO_2_ onto mtBTO NPs. The Zn content was further determined by inductively coupled plasma optical emission spectroscopy, and the results indicate that the weight percentage of ZnO_2_ in ZnO_2_@mtBTO NPs is 27.82 wt%. The hydrodynamic diameters of ZnO_2_@mtBTO NPs and SNO-ZnO_2_@mtBTO NPs are measured to be 229.8 nm and 267.2 nm, which are slightly larger than that of mtBTO NPs (197.6 nm) (Fig. 2e). The zeta potential of mtBTO, ZnO_2_@mtBTO, and SNO-ZnO_2_@mtBTO NPs were measured to be − 14.9, − 29.4, and − 35.1 mV, respectively (Fig. 2f). The grafting of SNO groups changes the solution appearance of ZnO_2_@mtBTO NPs from white to light yellow. In addition, successful grafting of SNO groups was also confirmed by the broadened optical absorption at UV–vis spectra (Fig. 2g). The characteristic peak of –N=O positioned at 1536 cm^−1^ on the FTIR spectrum was also indicated (Fig. 2h). The SNO grafting content was further quantified by NO detection kit after completely converting the SNO groups to NO by US stimulation, and the results indicate that 52.6 nmol SNO was incorporated into SNO-ZnO_2_@mtBTO NPs (with mtBTO weight of 25 mg).

The release of the encapsulated CDN could be facilitated by the tumor microenvironment-responsive degradation of ZnO_2_ under US irradiation. The decomposition kinetics of H_2_O_2_ could be unveiled using an H_2_O_2_ detection kit. The generation amount of H_2_O_2_ is proportional to NZCB NPs dose and pH conditions. Specifically, ZnO_2_@mtBTO NPs at doses of 62.5 μg/ml, 16.2 μmol/L, and 35.7 μmol/L of H_2_O_2_ could be generated respectively under neutral (pH = 7.4) and mildly acidic conditions (pH = 6.0) (Fig. 2i). We also observed gradual elevation of O_2_ production especially under acidic conditions with the dissolved oxygen meter. The O_2_ production amounts were quantified to be 33.9, 54.7, and 56.3 μmol/L at 500 μg/ml ZnO_2_@mtBTO NPs under varied pH conditions (7.4, 6.5, and 5.0) (Fig. 2j). Ion release was also verified by detecting the remaining Zn^2+^ and Ba^2+^ concentration of ZnO_2_@mtBTO NPs via inductively coupled plasma optical emission spectroscopy (Fig. 2k). It was found that the decomposition of ZnO_2_ reached 78.33 and 47.41% in nearly 24 h under pH 6.0 and 7.4 respectively. Such decomposition further releases the encapsulated CDN and Zn^2+^ for immune activations. Afterward, the loading amount and release behavior of CDN from NZCB NPs were evaluated by enzyme-linked immunosorbent assay (ELISA). For a typical initial mtBTO NPs dose of 50 μg/ml, the encapsulation efficiency of CDN is calculated to be 47%, and the loading amount of CDN was determined to be 0.235 mg within NZCB NPs (with mtBTO weight of 25 mg). Under neutral conditions (pH = 7.4), only 1.7% amount of CDN was released in 60 min. In contrast, a mildly acidic condition (pH = 6.0) was found to prompt the sustained release of CDN (10.1% in 60 min). Irradiation of US (1 MHz, 1.0 W/cm^2^, 50% duty ratio) to the solution (pH = 6.0) induces a significant increase of CDN released from NZCB NPs (41.0% released in 60 min), guaranteeing the stimuli-responsive drug release process (Fig. 2l).

### In vitro catalytic investigation of SNO-ZnO_2_@mtBTO NPs

**Fig. 3:**
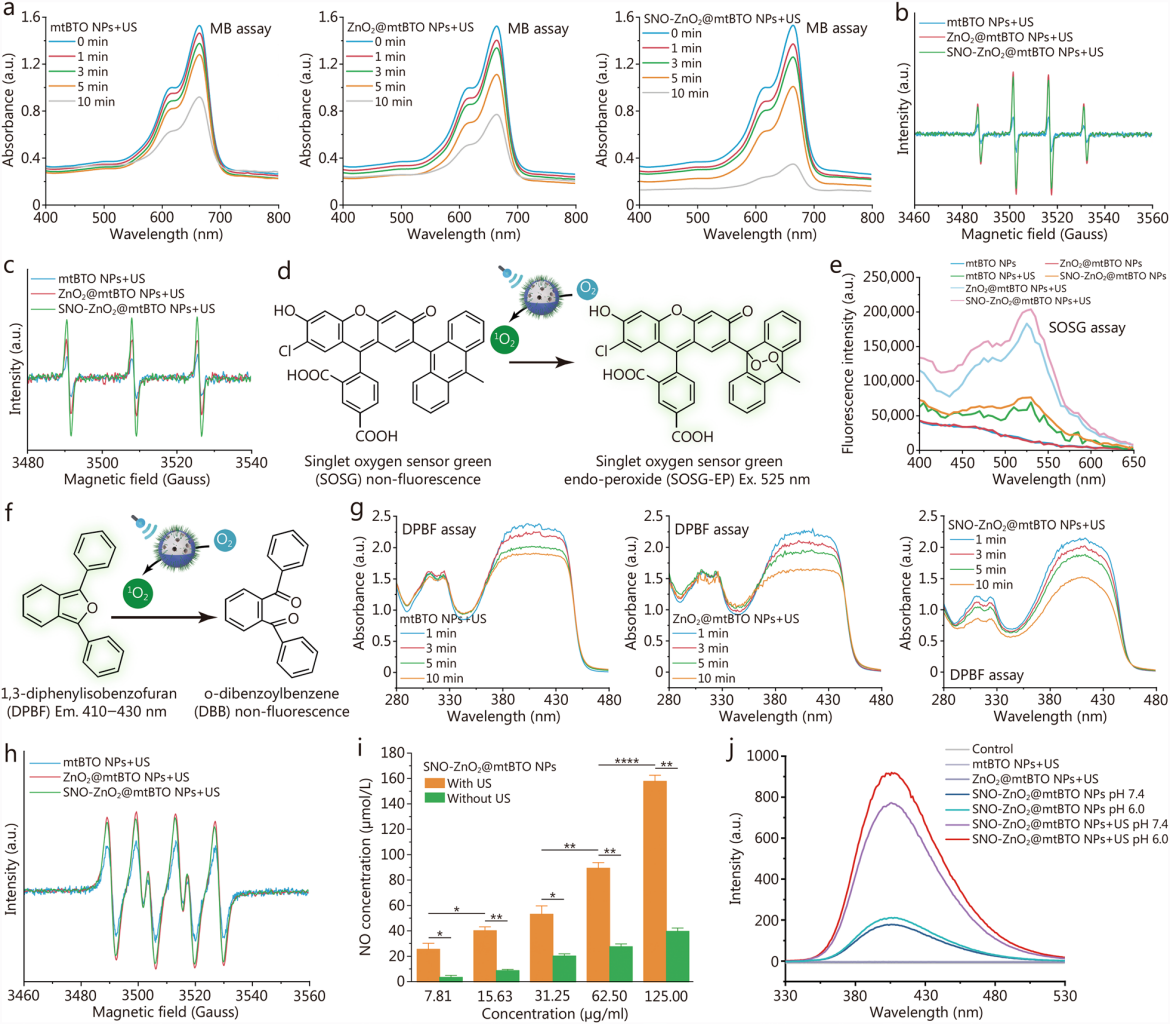
US-initiated cascade catalytic investigation of SNO-ZnO_2_@mtBTO NPs. **a** Optical absorbance curves of MB after co-incubation with mtBTO, ZnO_2_@mtBTO, and SNO-ZnO_2_@mtBTO NPs at 200 μg/ml after US irradiation (1.0 W/cm^2^) for 1, 3, 5, and 10 min under pH 7.4. ESR spectrum of BMPO/·OH (**b**) and BMPO/^1^O_2_ (**c**) after co-incubation with mtBTO, ZnO_2_@mtBTO, and SNO-ZnO_2_@mtBTO NPs at 20 μg/ml after US treatment (1.0 W/cm^2^, 1 min). **d** Mechanistic illustration of ^1^O_2_ detection via SOSG probe. **e** Fluorescence intensity of SOSG co-incubated with different NPs with or without US irradiation. **f** Mechanistic illustration of ^1^O_2_ detection via DPBF. **g** Absorbance curves of DPBF solution containing mtBTO NPs, ZnO_2_@mtBTO NPs and SNO-ZnO_2_@mtBTO NPs at 50 μg/ml after US treatment (1.0 W/cm^2^) for 1, 3, 5, and 10 min. **h** ESR spectrum of BMPO/·O_2_^−^ after co-incubation with mtBTO, ZnO_2_@mtBTO, and SNO-ZnO_2_@mtBTO NPs at 20 μg/ml after US treatment (1.0 W/cm^2^, 1 min). **i** NO production of SNO-ZnO_2_@mtBTO NPs with varied concentrations (7.81, 15.6, 31.3, 62.5 and 125.0 µg/ml). **j** Fluorescence curves of 3,3′-dityrosine in the system containing mtBTO, ZnO_2_@mtBTO, or SNO-ZnO_2_@mtBTO NPs at 20 μg/ml with or without US irradiation for ONOO^−^ detection under pH 7.4 and 6.0. *P < 0.05, **P < 0.01, ***P < 0.001, ****P < 0.0001. US ultrasound, MB methylene blue, ESR electron spin resonance, BMPO 5-tert-butoxycarbonyl-5-methyl-1-pyrroline-*N*-oxide, ·OH hydroxyl radical, O_2_ singlet oxygen radical, ·O_2_^*−*^ superoxide radical, SOSG singlet oxygen sensor green, DPBF 1,3-diphenylisobenzofuran, *SNO-ZnO2@mtBTO* S-nitrosothiols (SNO)-zinc peroxide (ZnO_2_)@mesoporous tetragonal barium titanate (mtBTO), NPs nanoparticles, Ex excitation, Em emission

SNO-ZnO_2_@mtBTO NPs are capable of generating a variety of ROS and RNS including H_2_O_2_, ·O_2_^−^, ^1^O_2_, ·OH, NO, and ONOO^−^ via cascade catalytic reactions. Initially, MB bleaching was employed to evaluate the total production of ROS and RNS via dye degradation. In the presence of US irradiation, mtBTO NPs could effectively generate ·O_2_^−^ and ·OH due to the piezoelectric performance, potentially bleaching MB in a time-dependent manner (Fig. 3a). When US irradiation was applied to ZnO_2_@mtBTO and SNO-ZnO_2_@mtBTO NPs, a substantial reduction of optical absorption at 664 nm could be indicated (Fig. 3a). For a regular US irradiation of 10 min (1.0 W/cm^2^, 50% duty ratio), the bleaching percentage of mtBTO, ZnO_2_@mtBTO, and SNO-ZnO_2_@mtBTO NPs could be calculated as 41.77, 49.36, and 77.23% respectively, implicating the prominent generation of the radical species. Acidic solution with pH 6.0 led to enhanced MB bleaching in ZnO_2_@mtBTO and SNO-ZnO_2_@mtBTO NPs, reaching the bleaching percentages of 61.04 and 79.34% respectively (Additional file 1: Fig. S14).

To identify the radical species generated during the piezoelectric catalytic process, we employed ESR to probe the formation of the radicals in the presence of nitrogen traps 5-tert-butoxycarbonyl-5-methyl-1-pyrroline-*N*-oxide. In the presence of US irradiation, we found that ZnO_2_@mtBTO and SNO-ZnO_2_@mtBTO NPs lead to the enhanced spectral amplitudes of 1:2:2:1 compared with mtBTO NPs, demonstrating the enhanced production of ^·^OH under US stimulation (Fig. 3b). We also observed the characteristic peak of 1:1:1 originated from ^1^O_2_ generated by ZnO_2_@mtBTO and SNO-ZnO_2_@mtBTO NPs, with higher spectral amplitudes in comparison with mtBTO NPs (Fig. 3c). The elevated generation of the ^1^O_2_ may be specifically attributed to the O_2_-producing ZnO_2_ from mtBTO NPs. The production of ^1^O_2_ was further quantified by a singlet oxygen sensor green (SOSG) probe, which forms fluorescent endo-peroxide structures after ^1^O_2_ exposure (Fig. 3d). ZnO_2_@mtBTO and SNO-ZnO_2_@mtBTO NPs were observed with a noticeable increase in SOSG fluorescence intensity after US irradiation (Fig. 3e). ^1^O_2_-specific probe DPBF was further used for ^1^O_2_ quantification (Fig. 3f). As the US irradiation time extends to 1, 3, 5, and 10 min, the fluorescence intensities of DPBF co-incubated with ZnO_2_@mtBTO NPs or SNO-ZnO_2_@mtBTO NPs gradually decreased (Fig. 3g). We also observed the characteristic peak of 1:1:1:1 originates from ·O_2_^−^ in ESR spectra. The spectral amplitude of ZnO_2_@mtBTO and SNO-ZnO_2_@mtBTO NPs was higher (Fig. 3h) in comparison with mtBTO NPs. Since ·O_2_^−^ radicals can easily react with NO, liberating ONOO^−^ with higher oxidative potentials and longer half-life, we further evaluated US-initiated cascade RNS generation. NO production was evaluated using the standard Griess method. When the Griess reagent was added to the solution containing SNO-ZnO_2_@mtBTO NPs of 50 µg/ml under US radiation with a 50% duty ratio for 1 min, the solution color gradually changed from light yellow to purple, indicating that US can trigger the release of NO from SNO-ZnO_2_@mtBTO NPs. Under US irradiation at 1.0 W/cm^2^, the released NO increased along with the SNO-ZnO_2_@mtBTO NPs concentration (7.81, 15.63, 31.25, 62.50, and 125.00 µg/ml) (Fig. 3i). Next, we employed the l-tyrosine-based fluorescence detection method for ONOO^−^ determination (ONOO^−^ can oxidize l-tyrosine to 3,3′-dityrosine, emitting fluorescence with excitation, emission wavelengths of 313, 406 nm. Co-incubation of l-tyrosine with SNO-ZnO_2_@mtBTO NPs solution containing saturated CO_2_ induced a weak fluorescent signal at 406 nm. The fluorescence intensity is significantly enhanced after US irradiation (1.0 W/cm^2^, 50% duty ratio) for 2 min, and was further enhanced under acid conditions due to accelerated ZnO_2_ decomposition (Fig. 3j). Collectively, the as-synthesized SNO-ZnO_2_@mtBTO NPs exhibit robust catalytic properties in the generation of oxidative species such as ·OH, ·O_2_^−^, ^1^O_2_, NO, and ONOO^−^ under US irradiations, potentially serving as the oxidative modulators against the tumor cells and tissues.

### In vitro SPDT against murine melanoma cells

**Fig. 4:**
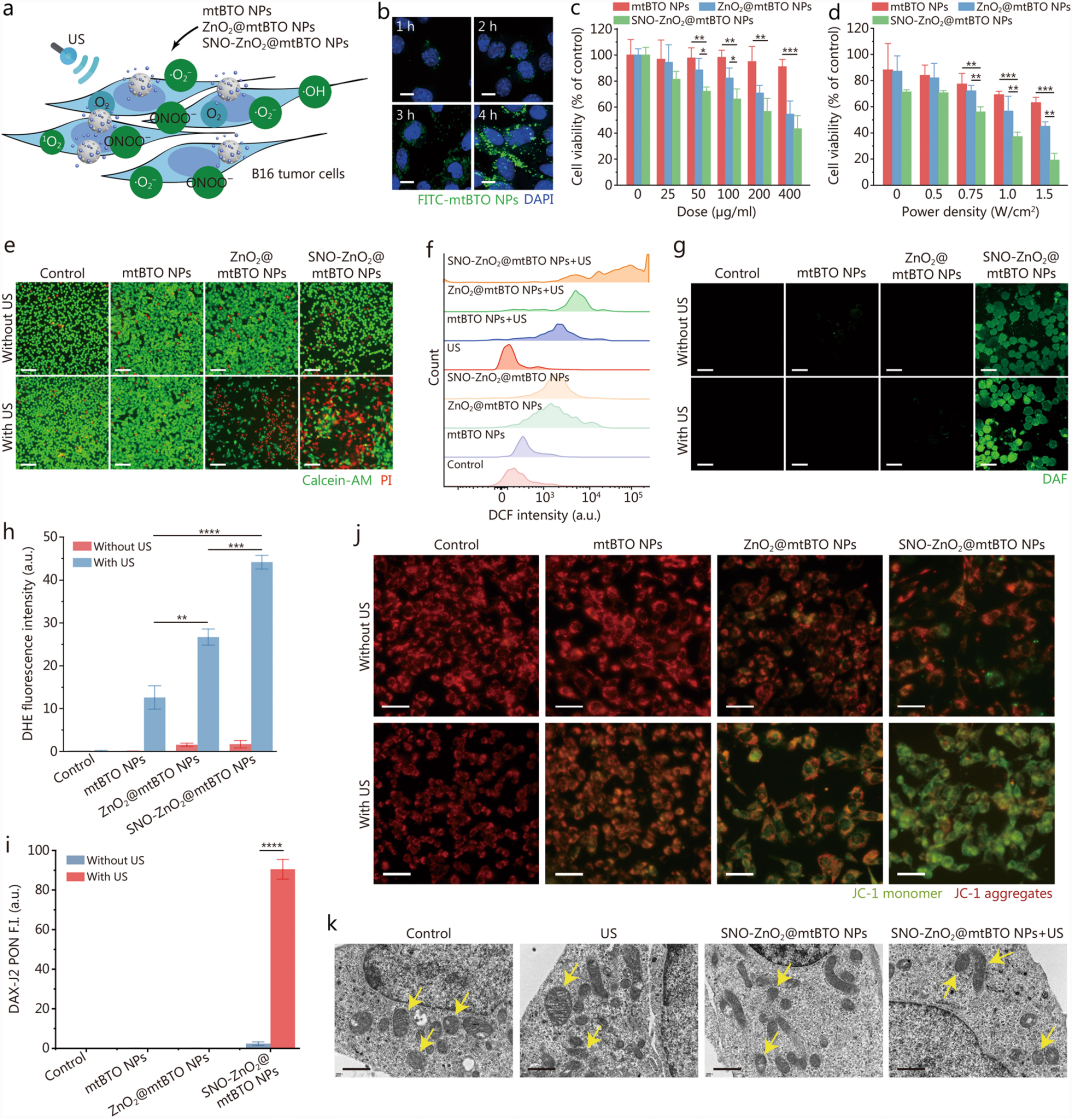
Intracellular sono-piezodynamic investigation of SNO-ZnO_2_@mtBTO NPs against B16 tumor cells. **a** Schematic illustration of US-initiated intracellular cascade catalytic effect by SNO-ZnO_2_@mtBTO NPs. **b** CLSM images of DAPI-stained B16 cells after co-incubation with FITC-mtBTO NPs at 50 μg/ml for 1, 2, 3 and 4 h (scale bar = 10 µm). **c** Cell viabilities of B16 cells after co-incubation with mtBTO, ZnO_2_@mtBTO, and SNO-ZnO_2_@mtBTO NPs at varied concentrations (0, 25, 50, 100, 200, and 400 μg/ml). **d** Cell viabilities of B16 cells after co-incubation with mtBTO, ZnO_2_@mtBTO, and SNO-ZnO_2_@mtBTO NPs at 50 μg/ml with US irradiation with varied power densities (0, 0.5, 0.75, 1.0, and 1.5 W/cm^2^). **e** Fluorescent microscopic images of Calcein-AM/PI-stained B16 cells after co-incubation with mtBTO, ZnO_2_@mtBTO, and SNO-ZnO_2_@mtBTO NPs at 50 μg/ml with or without US irradiation (1.0 W/cm^2^, 1 min) (scale bar = 50 μm). **f** Flow cytometry results of H_2_DCF-DA-stained B16 cells after co-incubation with mtBTO, ZnO_2_@mtBTO, and SNO-ZnO_2_@mtBTO NPs at 50 μg/ml with or without US irradiation (1.0 W/cm^2^, 1 min). **g** CLSM images of DAF-FM-stained B16 cells after co-incubation with mtBTO, ZnO_2_@mtBTO, and SNO-ZnO_2_@mtBTO NPs at 50 μg/ml with or without US irradiation (1.0 W/cm^2^, 1 min) (scale bar = 25 μm). Statistical analysis of fluorescence intensity of DHE− (**h**) or DAX-J2 PON Green-stained (**i**) B16 cells after co-incubation with mtBTO, ZnO_2_@mtBTO, and SNO-ZnO_2_@mtBTO NPs at 50 μg/ml with or without US irradiation (1.0 W/cm^2^, 1 min). **j** Fluorescent imaging of JC-1-stained B16 cells after co-incubation with mtBTO, ZnO_2_@mtBTO, and SNO-ZnO_2_@mtBTO NPs at 50 μg/ml with or without US irradiation (1.0 W/cm^2^, 1 min) (scale bar = 25 μm). **k** Bio-TEM images of B16 cells after co-incubation with mtBTO, ZnO_2_@mtBTO, and SNO-ZnO_2_@mtBTO NPs at 50 μg/ml with or without US irradiation (1.0 W/cm^2^, 1 min) (scale bar = 500 nm). *P < 0.05, **P < 0.01, ***P < 0.001, ****P < 0.0001. US ultrasound, CLSM confocal laser scanning microscopy, DAPI 4′,6-diamidino-2-phenylindole, FITC fluorescein isothiocyanate, Calcein-AM calcein-acetoxymethyl ester, PI propidium iodide, H_2_DCF-DA 2,7-dichlorodihydrofluorescein diacetate, DAF-FM 4-amino-5-methylamino-2,7-difluorofluorescein, DHE dihydroethidium, JC-1 5,5′,6,6′-tetrachloro-1,1′,3,3′-tetraethylbenzimidazolyl-carbocyanine iodide, Bio-TEM bio-transmission electron microscopy, SNO-ZnO_2_@mtBTO S-nitrosothiols (SNO)-zinc peroxide (ZnO_2_)@mesoporous tetragonal barium titanate (mtBTO), NPs nanoparticles

Inspired by the catalytic performance of US-excited piezoelectric cascades in vitro, we further explored the intracellular cascade catalytic effect of SNO-ZnO_2_@mtBTO NPs against murine melanoma B16 cell line (Fig. 4a). First, cellular uptake of mtBTO NPs was detected by CLSM. After co-incubating FITC-labeled mtBTO NPs with the cells for varied time points, intracellular FITC signals were observed after 1 h of co-incubation. The fluorescence intensity of FITC-mtBTO NPs increased over time. Notably, a significant accumulation of FITC-mtBTO NPs was observed after 4 h, indicating efficient uptake and substantial accumulation of mtBTO NPs by the cells (Fig. 4b). To avoid US-induced hyperthermia, the US power density and duty ratio are set to 1.0 W/cm^2^ and 50% respectively (Additional file 1: Fig. S15). US duration parameter was also optimized as 5 s (5 s on + 5 s off) to avoid cell detachment (Additional file 1: Fig. S16). Subsequently, we assessed the cytotoxic effects of the piezoelectric NPs on B16 tumor cells using a cell counting kit-8. It was found that, under identical US irradiation conditions (1.0 W/cm^2^, 1 min), the B16 cell survival rate decreased as the increased co-incubation doses of mtBTO, ZnO_2_@mtBTO, or SNO-ZnO_2_@mtBTO NPs (Fig. 4c). Additionally, at the same concentrations of different NPs (50 µg/ml), we observed a decrease in the cell survival rate of B16 cells as the power density of US increased (Fig. 4d). The decrease in cell survival rate in the SNO-ZnO_2_@mtBTO NPs following US treatment was notably evident. Subsequently, we conducted an assessment of biocompatibility and performed live/dead staining (calcein-acetoxymethyl ester/propidium iodide) for B16 cells co-incubated with indicated treatments. It was observed that a majority of B16 cells exhibit green fluorescence after co-incubation of 50 µg/ml mtBTO, ZnO_2_@mtBTO, or SNO-ZnO_2_@mtBTO NPs in the absence of US irradiation. When the US was supplemented to the cells, only mtBTO NPs-treated cells maintained the fluorescence intensity of the live cells. For ZnO_2_@mtBTO and SNO-ZnO_2_@mtBTO NPs in the presence of US (1.0 W/cm^2^, 1 min), a major proportion of the cells died, as revealed by the red fluorescence of the confocal images (Fig. 4e).

Cellular ROS and RNS production were then evaluated via the fluorescence probe 2,7-dichlorodihydrofluorescein diacetate, which responsively reacts with oxidative substances to produce green fluorescence. According to the confocal microscopic observations, dichlorodihydrofluorescein fluorescence could be detected in the cells from ZnO_2_@mtBTO and SNO-ZnO_2_@mtBTO NPs groups. US irradiation (1.0 W/cm^2^, 1 min) of the cells augments the intracellular production of the oxidative species, brightening the fluorescence intensities of the cells treated with ZnO_2_@mtBTO and SNO-ZnO_2_@mtBTO NPs (Additional file 1: Figs. S17 and S18). The flow cytometry results also confirm the enhanced fluorescence intensity of the cells treated with ZnO_2_@mtBTO and SNO-ZnO_2_@mtBTO NPs under US irradiation (Fig. 4f). To investigate the oxidative species specifically, we employed fluorescence probes with radical specificity for intracellular evaluation [SOSG for ^1^O_2_, 4-amino-5-methylamino-2,7-difluorofluorescein for NO, dihydroethidium (DHE) for ·O_2_^−^, and DAX-J2 for ONOO^−^]. Consistent with the quantification results at the solution level, intracellular SOSG fluorescence signals were observed in the cells treated with ZnO_2_@mtBTO or SNO-ZnO_2_@mtBTO NPs, especially with US supplementation (Additional file 1: Fig. S19). Typically, SNO-ZnO_2_@mtBTO NPs induce prominent diaminofluorescein fluorescence. US-promoted NO production was also verified by the enhanced diaminofluorescein fluorescence in the cells from the SNO-ZnO_2_@mtBTO NPs + US group (Fig. 4g). DHE fluorescence was also gradually enhanced for cells treated with mtBTO, ZnO_2_@mtBTO, and SNO-ZnO_2_@mtBTO NPs, specifically with US supplementation (Fig. 4h; Additional file 1: Fig. S20). Intracellular ONOO^−^ production was confirmed by the statistically enhanced fluorescence intensity of DAX-J2 in the SNO-ZnO_2_@mtBTO NPs + US group, indicating the highest quantities of ONOO^−^ radicals originated from the piezoelectric catalytic cascade (Fig. 4i; Additional file 1: Fig. S21).

Mitochondria is the most dominant target of most oxidative species. We then evaluate the status of mitochondrial polarization using JC-1 probes. The B16 cells treated with SNO-ZnO_2_@mtBTO NPs in the presence of US significantly increase the green fluorescence of J aggregates, indicating the increased membrane permeability and consequent loss of electrochemical potential during cellular apoptosis (Fig. 4j; Additional file 1: Fig. S22). Apoptotic morphological changes in the subcellular organelles of B16 tumor cells with SNO-ZnO_2_@mtBTO NPs + US treatment were observed on Bio-TEM images (Fig. 4k). Apoptotic characteristics such as mitochondrial swelling, endoplasmic reticulum expansion, vacuolization and fracture could also be identified. These evidences collectively demonstrate the piezoelectric SNO-ZnO_2_@mtBTO NPs could responsively burst a variety of oxidative radicals to trigger mitochondrial damage and cell apoptosis, presenting great promises for tumor destruction based on cascade piezoelectric catalysis.

### SNO-ZnO_2_@mtBTO NPs activate tumor PANoptosis by nanocatalytic oxidative strategy

**Fig. 5:**
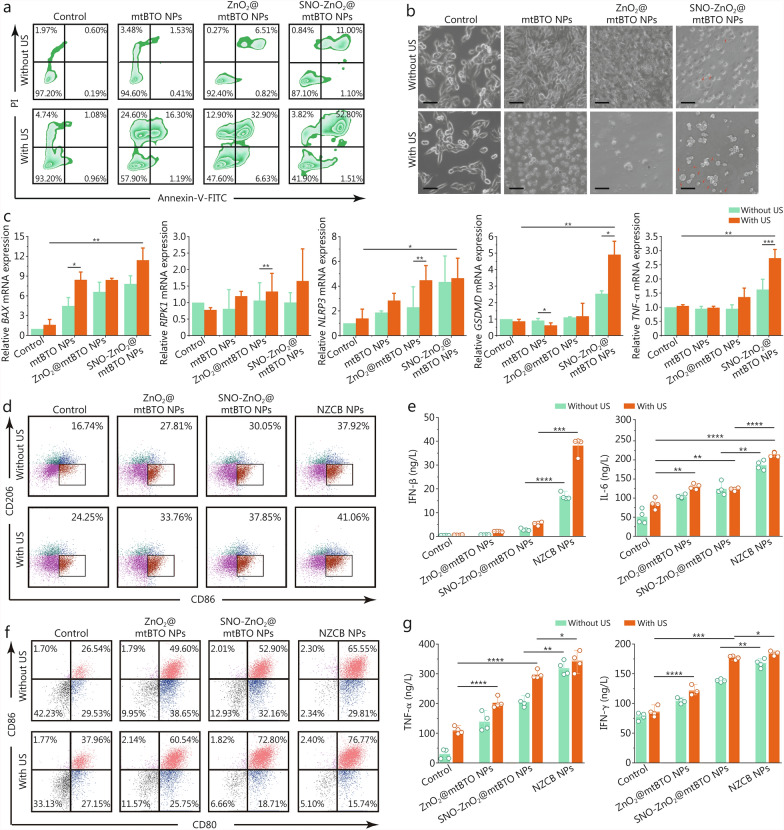
NZCB NPs activates immunogenic tumor PANoptosis by nanocatalytic oxidation strategy. **a** Flow cytometry analysis of Annexin V-FITC/PI-stained B16 cells treated with PBS, mtBTO NPs, ZnO_2_@mtBTO NPs, and SNO-ZnO_2_@mtBTO NPs (50 μg/ml) with or without US irradiation (1.0 W/cm^2^, 1 min). **b** Optical microscopic images of B16 cells treated with PBS, mtBTO NPs, ZnO_2_@mtBTO NPs, and SNO-ZnO_2_@mtBTO NPs (50 μg/ml) with or without US irradiation (1.0 W/cm^2^, 1 min) (scale bar = 20 μm). **c** RT-qPCR analysis of *BAX*, *RIPK1*, *NLRP3*, *GSDMD*, and *TNF-α* expression in B16 cells treated with PBS, mtBTO NPs, ZnO_2_@mtBTO NPs, and SNO-ZnO_2_@mtBTO NPs (50 μg/ml) with or without US irradiation (1.0 W/cm^2^, 1 min). Flow cytometry analysis (**d**), and corresponding IFN-β and IL-6 ELISA results (**e**) of the supernatant of RAW264.7 cells treated with PBS, ZnO_2_@mtBTO NPs, SNO-ZnO_2_@mtBTO NPs, and NZCB NPs (50 μg/ml) with or without US irradiation (1.0 W/cm^2^, 1 min). Flow cytometry analysis (**f**), and corresponding TNF-α and IFN-γ ELISA results (**g**) of the supernatant of BMDCs after incubating with B16 tumor cells which were pretreated with PBS, ZnO_2_@mtBTO NPs, SNO-ZnO_2_@mtBTO NPs, and NZCB NPs (50 μg/ml) with or without US irradiation (1.0 W/cm^2^, 1 min). *P < 0.05, **P < 0.01, ***P < 0.001, ****P < 0.0001. Annexin V-FITC PI propidium iodide, RT-qPCR quantitative reverse transcription polymerase chain reaction, BAX Bcl-2 associated X protein, RIPK1 receptor interacting serine/threonine kinase 1, NLRP3 nucleotide oligomerization domain-like receptor pyrin domain containing 3, GSDMD gasdermine-D, TNF-α tumor necrosis factor-α, IFN-β interferon-β, IL-6 interleukin-6, ELISA enzyme-linked immunosorbent assay, NZCB S-nitrosothiols (SNO)-zinc peroxide (ZnO_2_)@cyclic dinucleotide (CDN)@mesoporous tetragonal barium titanate (mtBTO), US ultrasound, BMDCs bone marrow dendritic cells

We next gain deeper insights into the cell death pathways induced by the piezoelectric cascade catalytic reactions. Initially, Annexin V-FITC/PI-based flow cytometry analysis was conducted for B16 tumor cells subjected to various treatments. Specifically, mtBTO NPs induce 24.6% of necrotic cells (Annexin V-FITC-/PI+) and 16.3% of late apoptotic cells (Annexin V-FITC+/PI+) with US irradiation. For ZnO_2_@mtBTO NPs + US or SNO-ZnO_2_@mtBTO NPs + US treatment, the proportions of necrotic cells decreased and cells with late apoptosis increased, suggesting the potential co-occurrence of apoptosis and necroptosis (Fig. 5a). From optical observations, cell apoptosis could be confirmed for cells treated with mtBTO NPs + US and ZnO_2_@mtBTO NPs + US groups. For cells from SNO-ZnO_2_@mtBTO NPs + US group, apoptotic bodies and pyroptotic inflammasomes were observed, collectively implicating the mix of cell death pathways including necroptosis, apoptosis, and pyroptosis (Fig. 5b). We then evaluated the predominant mRNA levels that coordinate these cell death pathways using RT-qPCR. The evaluated mRNAs (*BAX*, *RIPK1*, *NLRP3*, *GSDMD,* and *TNF-α*) exhibited elevated expression levels, particularly with SNO-ZnO_2_@mtBTO NPs + US treatment (Fig. 5c). These results preliminarily indicate that the constructed piezoelectric SNO-ZnO_2_@mtBTO NPs could effectively induce multiple cell death pathways (necroptosis, apoptosis, and pyroptosis) against B16 tumor cells under US irradiation, corresponding to the pathological characteristics of PANoptosis.

### NZCB NPs induce macrophage polarization and DC maturation in vitro

NZCB NPs with CDN encapsulation further enhances anti-tumor immunity since it could be co-localized in DCs or macrophages with the tumor antigen induced by SPDT, further activates the STING signaling. Therefore, in vitro macrophage polarization profile was firstly evaluated on murine macrophage RAW264.7 cell line. Initially, the biocompatibility of NZCB NPs on RAW264.7 cells was carried out. We found that NZCB NPs at doses less than 50 μg/ml and in the presence of US at power densities less than 1.0 W/cm^2^ show higher biocompatibility to RAW264.7 cells than the B16 tumor cells (Additional file 1: Fig. S23). For macrophage phenotype identification, a major population of the untreated cells exhibited M0 phenotype (42.7% CD86^−^CD206^−^). When these cells were treated with NZCB NPs with or without US irradiation, the M0 phenotype was polarized to M1 phenotype (CD86^+^CD206^−^) specifically, with the population quantified to be 37.92 and 41.06% respectively (Fig. 5d). Furthermore, RAW264.7 cells were incubated with B16 tumor cells, which were pretreated with PBS, ZnO_2_@mtBTO NPs, SNO-ZnO_2_@mtBTO NPs, and NZCB NPs with or without US irradiations through transwell setup. The results further identified M1 polarization from 8.41% in the control group to 39.22% after NZCB NPs + US treatment of B16 tumor cells (Additional file 1: Fig. S24), indicating the enhanced M1 polarization of the macrophages stimulated by the SPDT-enabled cell-killing effect synergized with the enhanced STING activity contributed by the released Zn^2+^ and the agonist CDN [33, 34]. In addition, the proinflammatory cytokines [such as interferon-β (IFN-β) and interleukin-6 (IL-6)] secreted from RAW264.7 after indicated treatments were found to be elevated, confirming the activation of STING pathway and macrophage polarization towards the M1 phenotype (Fig. 5e).

Serving as a representative role during adaptive immune responses, the maturation of the BMDCs was also evaluated. Using the transwell setup, BMDCs isolated from the bone marrow of mice were placed in the lower chamber, and the B16 cells after co-incubation with ZnO_2_@mtBTO, SNO-ZnO_2_@mtBTO, and NZCB NPs with or without US irradiations, were placed in the upper chamber. It was observed that BMDC maturation induced by SNO-ZnO_2_@mtBTO NPs increased from 52.90 to 72.80% before and after US irradiation (Fig. 5f), confirming US-initiated oxidative bursts for cell death. Furthermore, we found that NZCB NPs treatment induced a higher maturation population of BMDCs, possibly due to the release of CDN for STING activation. We also conducted ELISA of the supernatant of the BMDCs to further identify elevated excretion of inflammatory cytokines including tumor necrosis factor-α (TNF-α) and IFN-γ (Fig. 5g), collectively demonstrating the oxidative inflammation within the tumor cells.

### In vivo US-initiated SPDT and synergistic immunotherapy

**Fig. 6:**
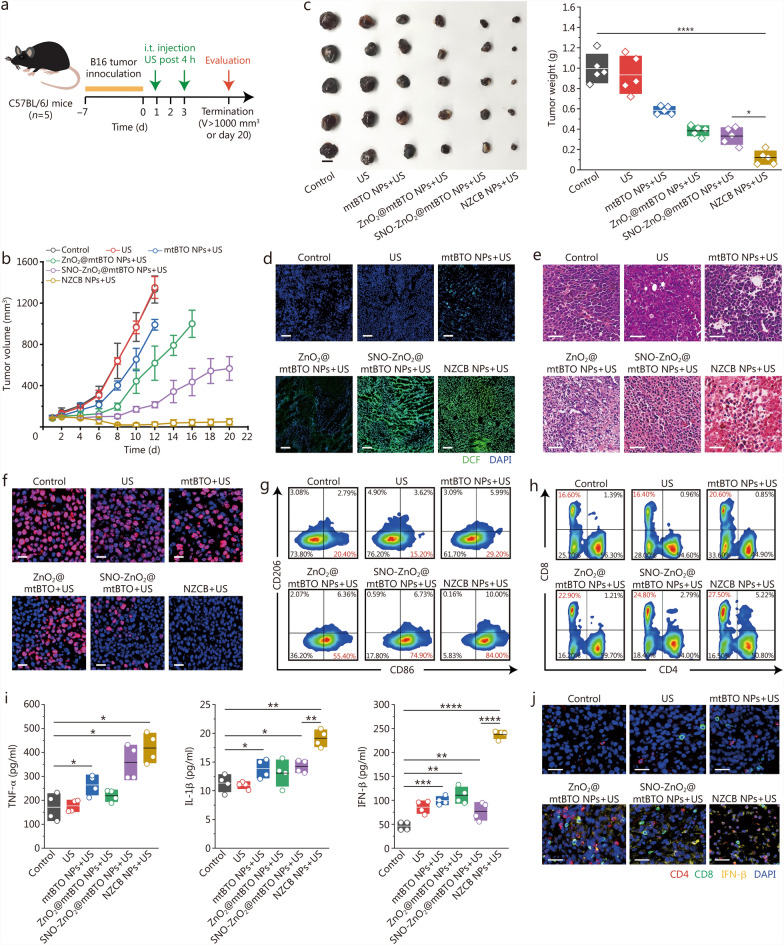
In vivo US-initiated sono-piezodynamic therapy (SPDT) and nanocatalytic immunopotentiation. **a** Treatment schedule of in vivo US-initiated SPDT on C57BL/6 J mice. **b** Tumor volume curves of mice from different groups during the evaluation timeframe. **c** Digital photographs and weight statistics of excised mouse tumors from different groups at the end of the evaluation. **d** Fluorescent microscopic images of H_2_DCF-DA-stained tumor sections of mice from different groups (scale bar = 100 μm). Microscopic images of H&E stained (**e**, scale bar = 50 μm) and antigen Ki-67 stained (**f**, scale bar = 20 μm) tumor sections of mice from different groups. Representative flow cytometry analysis of tumor-infiltrating immune cells, including macrophages (marked with CD86 and CD206) (**g**) and T cells (marked with CD4 and CD8) (**h**) isolated from mice in different groups. **i** Expression levels of TNF-α, IL-1β and IFN-β in mice serum extracted from mice in different groups, tested with ELISA. **j** Merged immunofluorescence staining of CD4, CD8, and IFN-β in ultrathin tumor sections of mice from different groups (scale bar = 50 μm). *P < 0.05, **P < 0.01, ***P < 0.001, ****P < 0.0001. H_2_DCF-DA 2,7-dichlorodihydrofluorescein diacetate, H&E hematoxylin and eosin, Ki-67 nuclear-associated antigen Ki-67, TNF-α tumor necrosis factor-α, IL-1β interleukin-1β, IFN-β interferon-β, NZCB S-nitrosothiols (SNO)-zinc peroxide (ZnO_2_)@cyclic dinucleotide (CDN)@mesoporous tetragonal barium titanate (mtBTO), US ultrasound

We next conducted further in vivo experiments on subcutaneous B16 tumor-bearing C57BL/6J to explore the therapeutic efficacy of NZCB NPs. Thirty mice were firstly divided into 6 groups: control, US, mtBTO NPs + US, ZnO_2_@mtBTO NPs + US, SNO-ZnO_2_@mtBTO NPs + US, and NZCB NPs + US. B16 cells (10^6^ cells dispersed in 100 μl saline) were subcutaneously injected into the right flank of each mouse. The tumor xenografts were allowed to develop to an average tumor volume of approximately around 90 mm^3^, before the initiation of the treatment. On day 1 and day 3, corresponding NPs were intratumorally injected into the xenografts (10 mg/kg dispersed in 100 μl saline). US irradiation was conducted (1 MHz, 1.0 W /cm^2^ for 3 min, 50% duty ratio) 4 h post-injection of the NPs to initiate the SPDT (Fig. 6a).

The body weight of mice was recorded every 2 d and no obvious weight loss was found after different treatments (Additional file 1: Fig. S25). The therapeutic efficiency was evaluated via the tumor growth curve. The control group and US group were observed with exponential growth of the xenografts. Mild tumor suppression effect could be observed for xenograft of mice from mtBTO NPs + US group, due to the US-triggered SPDT. For mice of ZnO_2_@mtBTO NPs + US and SNO-ZnO_2_@mtBTO NPs + US groups, the growth of the tumor xenografts was substantially inhibited, implicating the augmented SPDT outcome. Nevertheless, the therapeutic effect against the xenografts decayed on day 12, resulting in the uncontrolled proliferation of the tumor xenograft. For mice treated with NZCB NPs + US, all tumor xenografts were substantially destroyed within the whole evaluation timeframe. The endpoint tumor volume of the xenografts of mice from different groups was determined to be 1331 mm^3^ (day 12), 1351 mm^3^ (day 12), 989 mm^3^ (day 12), 999 mm^3^ (day 16), 566 mm^3^ (day 20), and 48 mm^3^ (day 20) respectively, validating the prominent US-initiated SPDT and catalytic anti-tumor immunities (Fig. 6b; Additional file 1: Fig. S26). At the end of the evaluation timeframe, all mice were recorded with digital tumor images (Additional file 1: Fig. S27) and then sacrificed with major organs and xenografts dissected. The digital photographs and weights of the xenografts directly confirm the robustly enhanced tumor suppression (0.996 g for the control group and 0.12 g for the NZCB + US group) via cascade SPDT synergized with the activated antitumor immunities (Fig. 6c).

To visualize the intratumoral distribution of the oxidative species, 2,7-dichlorodihydrofluorescein diacetate fluorescence probe was employed to stain the ultrathin section of tumors of mice from different groups. Significantly enhanced dichlorodihydrofluorescein fluorescence intensity could be observed for the xenografts from SNO-ZnO_2_@mtBTO NPs + US group and NCZB NPs + US group, confirming the intratumoral burst of the oxidative species enabled by SNO-ZnO_2_@mtBTO or NCZB NPs in the presence of US (Fig. 6d). Afterward, histopathological staining was performed to evaluate the therapeutic effects. Haematoxylin and eosin staining of the tumor sections demonstrate severe tumor cell damage and apoptosis, particularly in the NZCB + US group (Fig. 6e). Terminal-deoxynucleotidyl transferase-mediated nick end labeling staining reveals a pro-apoptotic effect on the tumor cells after NZCB NPs + US treatment (Additional file 1: Fig. S28). Similarly, the antigen Ki-67 antibody immunofluorescence staining indicates a decrease in proliferation populations of tumor cells in the NZCB + US group (Fig. 6f). Therapeutic biocompatibility was also investigated through histopathological staining of the major organs (heart, liver, spleen, lung, kidney) of mice from different groups. From these microscopic images, no pathological abnormalities could be revealed (Additional file 1: Fig. S29). Blood samples were also collected for hematological analysis at the end of treatments. All blood routine analyses and biochemical indexes were maintained within the normal ranges, supporting therapeutic biocompatibility and biosafety (Additional file 1: Fig. S30). In vivo biodistribution of the NZCB NPs after intratumoral injection was also evaluated. The results indicate a major intratumoral accumulation of 39.91 ID%/g in 48 h post-injection, compared to the lower biodistribution in liver (7.82 ID%/g) and spleen (2.51 ID%/g) due to the blood circulation by lymphatic drainage (Additional file 1: Fig. S31).

The activation of tumor-associated macrophages was evaluated, in which the M1-phenotype polarized macrophage (F4/80^+^CD86^+^CD206^−^) percentage increased from 20.40% in the control group to 84.00% in the NZCB + US group (Fig. 6g; Additional file 1: Fig. S32). The population and phenotypic characterization of tumor-infiltrating lymphocytes were then evaluated via CD3, CD4, and CD8 antibodies incubation with isolated lymphocytes from spleen tissues. Flow cytometry analysis revealed that CD4^+^CD8^+^ T cells increased from 1.39% (control group) to 5.22% (NZCB + US group), while for CD4^−^CD8^+^ T cells, the population markedly increased from 16.60% (control group) to 27.50% (NZCB + US group), indicating the activated anti-tumor T cell immunity (Fig. 6h; Additional file 1: Fig. S33). Statistical analysis of CD86^+^ macrophages and CD8^+^ T cells also validated the elevated M1 macrophages and cytotoxic T cells, indicating in vivo SPDT-triggered antitumoral immunities (Additional file 1: Fig. S34). Besides, typical proinflammatory cytokines in serum including TNF-α, IL-1β, and IFN-β were also detected using the ELISA. The increased secretion of TNF-α and IL-1β originates from immune activation and localized intratumoral inflammation by piezoelectric NCZB NPs-initiated SPDT (Fig. 6i). Type-I IFN-β secretion verifies strong stimulation of the STING signaling pathway by the constructed NZCB NPs in the presence of US (Fig. 6i). Consistently, microscopic images of the immunofluorescence staining demonstrated that the population of CD4^+^, CD8^+^ tumor-infiltrating lymphocytes and IFN-β expression were markedly enhanced via US-initiated SPDT synergized with STING agonists (Fig. 6j).

### Mechanism of tumor PANoptosis and STING immunity activation induced by NZCB NPs

**Fig. 7:**
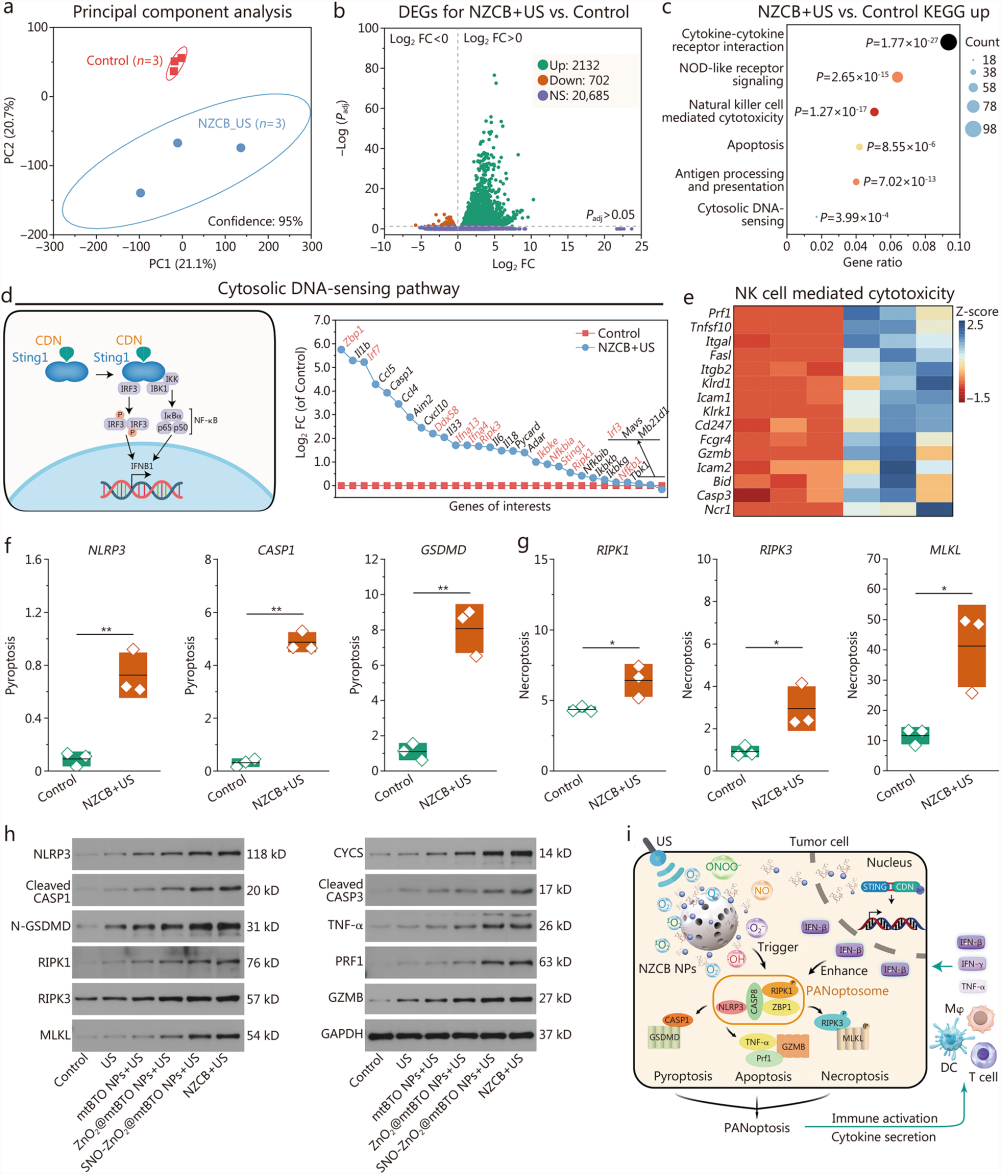
NZCB NPs induce PANoptosis and their genetic alterations. **a** Principal component analysis results of mRNA expressions between the control and NZCB + US groups. **b** Volcano plot of DEGs between the control and NZCB + US groups. **c** KEGG enrichment analysis of DEGs between the control and NZCB + US groups. **d** Schematic illustration of STING pathway and corresponding log_2_FC values of the DEGs between the control and NZCB + US groups. **e** Heat map of DEGs in NK cell cytotoxicity pathway between the control and NZCB + US groups. Expression profiles of pyroptosis-associated genes including *NLRP3*, *CASP1*, *GSDMD* (**f**) and necroptosis-associated genes including *RIPK1*, *RIPK3*, and *MLKL* (**g**). **h** Western blotting analysis of PANoptosis-related proteins of NLRP3, cleaved CASP1, N-GSDMD, RIPK1, RIPK3, MLKL, CYCS, cleaved CASP3, TNF-α, PRF1, GZMB, and GAPDH. **i** Schematic illustration of tumor PANoptosis pathway and immune activation. *P < 0.05, **P < 0.01. NZCB S-nitrosothiols (SNO)-zinc peroxide (ZnO_2_)@cyclic dinucleotide (CDN)@mesoporous tetragonal barium titanate (mtBTO), NPs nanoparticles, US ultrasound, DEGs differentially expressed genes, KEGG Kyoto Encyclopedia of Genes and Genomes, STING stimulator of interferon genes, Log_2_ FC Log_2_ (fold change), NK natural killer (cells), NLRP3 NLR family pyrin domain containing 3, CASP1 caspase-1, GSDMD gasdermin D, RIPK1 receptor-interacting serine/threonine-protein kinase 1, RIPK3 receptor-interacting serine/threonine-protein kinase 3, MLKL mixed lineage kinase domain-like (protein), CYCS cytochrome c, CASP3 caspase-3, TNF-α tumor necrosis factor-α, PRF1 perforin-1, GZMB granzyme B, IFN-β interferon-β, ZBP1 Z-DNA binding protein 1, DCs dendritic cells

To elucidate the genetic alteration mechanism by which NZCB NPs + US induces tumor cell death at the transcriptional level, a comprehensive transcriptome analysis was conducted on B16 tumor xenografts at the end of treatments using mRNA sequencing. The analysis encompasses untreated tumor xenografts (control) and xenografts exposed to NZCB NPs in the presence of US (NZCB + US). Within the entire transcriptome dataset, unsupervised dimensionality reduction was performed via principal component analysis on all differentially expressed genes (DEGs), including PC1 and PC2, jointly accounting for 41.8% of the variation (Fig. 7a). Notably, principal component analysis effectively discriminated between the control group and the NZCB + US group, underscoring substantial transcriptional disparities between these 2 cohorts. These preliminary findings suggest that NZCB NPs + US exhibits anti-tumor effects at the mRNA transcriptional level. The distribution of genes in the 2 treatment groups is depicted in the volcano plot for pairwise comparisons (Fig. 7b). In comparison to the untreated group, the NZCB + US group displayed 2132 significantly upregulated genes, 702 significantly downregulated genes, and 20,685 genes with no significant alterations. Among the DEGs identified in B16 xenografts after NZCB NPs + US treatment, Kyoto Encyclopedia of Genes and Genomes (KEGG) pathways including cytokine-cytokine receptor interaction, nucleotide-binding oligomerization domain-like receptor signaling, natural killer (NK) cell mediated cytotoxicity, apoptosis, antigen processing and presentation, and cytosolic DNA-sensing were significantly enriched (with a *P*-value cutoff of 0.05) (Fig. 7c).

Specifically, mRNAs of DEGs such as Z-DNA binding protein 1 (*Zbp1*), leucine-rich repeat flightless-interacting protein 7 (*Lrf7*), DEAD (Asp-Glu-Ala-Asp) box polypeptide 58 (*Ddx58*), interferon alpha 13 (*Ifna13*), *Ifna4*, receptor-interacting serine/threonine-protein kinase 3 (*Ripk3*), inhibitor of nuclear factor kappa-B kinase subunit epsilon (*Ikbke*), nuclear factor kappa B inhibitor alpha (*Nfkbia*), stimulator of interferon genes 1 (*Sting1*), *Ripk1*, nuclear factor kappa B subunit 1 (*Nfkb1*), and interferon regulatory factor 3 (*Irf3*) were found with significant upregulation after NZCB NPs + US treatment (Fig. 7d). These mRNAs were allocated with the cytosolic DNA-sensing KEGG pathway, suggesting that the released CDN successfully activated the STING antitumoral immunity. We also notice that the upregulation of the NK cell cytotoxicity has been enriched. As compared to the control group, mRNAs of DEGs [tumor necrosis factor superfamily member 10 (*Tnfsf10*), Fas ligand (*Fasl*), killer cell lectin like receptor D1 (*Klrd1*), granzyme B (*Gzmb*), natural cytotoxicity triggering receptor 1 (*Ncr1*), and perforin 1 (*Prf1*)] that coordinate the innate antitumoral immunity have been differentially upregulated, positively associated with target cell recognition*,* granule exocytosis and cell killing of innate immune cells (Fig. 7e). The enriched KEGG pathways also record the involvement of cell pyroptosis, apoptosis, and necroptosis, revealing the PANoptosis feature induced by NZCB NPs in the presence of US. To validate the cell death pattern in comparison to the control group, the NZCB + US group exhibited elevated levels of NLRP3, CASP1, and GSDMD, which are positively associated with pyroptosis (*P* < 0.05) (Fig. 7f). For apoptosis-associated mRNAs, upregulated genes typically include *BAX*, *CYCS*, *CASP3*, *TNF-α*, *PRF1*, and *GZMB* (Additional file 1: Fig. S35). Necroptosis-associated genes such as *RIPK1*, *RIPK3*, and mixed lineage kinase domain-like protein (*MLKL*) were also significantly upregulated in the NZCB + US group as compared to the control group (*P* < 0.05) (Fig. 7g). These proteins were further verified using Western blotting analysis between tumor samples treated with control, US, mtBTO NPs + US, ZnO_2_@mtBTO NPs + US, SNO-ZnO_2_@mtBTO NPs + US and NZCB NPs + US. NLRP3, cleaved CASP1 and N-GSDMD, were notably enhanced, especially after NZCB NPs + US treatment. The elevated expressions of CYCS and cleaved CASP3 indicate intrinsic apoptosis, while the upregulated expressions of TNF-α, PRF1, and GZMB indicate the occurrence of extrinsic apoptosis. In addition, the higher expression of RIPK1, RIPK3, and MLKL implicates tumor necroptosis (Fig. 7h; Additional file 1: Fig. S36). However, the specific synergistic mechanism of oxidative stress and STING immunity in inducing PANoptosis remains unclear. We hypothesize that the STING agonist activates the expression of IRF1 and further induces the activation of ZBP1, which is a key signaling protein proposed previously [36]. In addition, oxidative stress activates CASP8 via the Fas and FasL pathway (Additional file 1: Fig. S37), ultimately leading to the assembly of ZBP1-CASP8 PANoptosome and PANoptosis of B16 tumor cells. Collectively, the US-initiated SPDT enabled by NZCB NPs effectively triggered tumor-specific pyroptosis, apoptosis, and necrosis (PANoptosis) during in vivo tumor therapy against the melanoma cells, synergistically promoting both innate and adaptive immunities for tumor catalytic immunotherapy (Fig. 7i).

## Discussion

Compared to reported studies using non-porous BTO NPs [35], this work reported the invention of the boiling-bubbling method to transform the spherical non-porous BTO NPs into mesoporous architecture, facilitating the delivery of small molecules, mRNAs, plasmids, or proteins for diverse biomedical applications with significance. Meanwhile we successfully induced PANoptosis of tumor cells through the bursts of oxidative species and activation of STING immunity. Notably, at the cellular level, we observed necroptosis as the primary cell death pattern, accompanied by cell pyroptosis. However, during in vivo investigation, the proportion of pyroptosis was significantly enhanced. This discrepancy may be attributed to the potential in vivo immune responses to CDN-mediated STING immunity. The release of Zn^2+^ and CDN creates a robust inflammatory environment intratumorally, which could synergize with the oxidative species to induce potent PANoptosis of tumor cells. Furthermore, during the transcriptomic study, endogenous apoptosis-related genes such as *CYCS* were not upregulated, while exogenous apoptosis-related *TNF*, *PRF1*, and *GZMB* were significantly upregulated, resulting from the activation of cytotoxic T lymphocytes and NK cells for cell killing. We also demonstrated that oxidative stress activates CASP8, while STING agonist upregulates IRF1 expression, subsequently inducing ZBP1 and coordinating ZBP1-CASP8 PANoptosome formation in tumor cells. Research on detail regulation mechanisms is still in progress. In conclusion, we have demonstrated that nanocatalytic oxidative stress by reactive oxygen and nitrogen species plays an indispensable role in triggering tumor PANoptosis. Finally, with the development of medical US in the application of imaging, NZCB-based SPDT is particularly appealing for clinically transformable tumor therapeutics, especially for skin melanoma and other superficial tumors such as breast carcinoma.

## Conclusions

In conclusion, we have presented a novel chemical approach to fabricate controllable and customizable mtBTO NPs for CDN encapsulation and SNO-functionalized ZnO_2_ decoration, forming NCZB NPs for US-initiated SPDT. Under the irradiation of US, NCZB NPs are capable of bursting quantities of ROS (·OH, ·O_2_^−^, ^1^O_2_) and RNS (NO, ONOO^−^) responsively, inducing prominent oxidative stresses both intracellularly and intratumorally. The on-demand release of CDN could activate the STING immunity, which was further enhanced by the released Zn^2+^, remodeling the microenvironment to be immune-activated for tumor cell destruction. NCZB NPs have constructed the oxidative inflammatory milieu to induce nanocatalytic tumor PANoptosis with immunopotentiation, ultimately destroying the tumor tissues with high effectiveness and biocompatibility.

## Supplementary Information

**Additional file 1.** Methods. **Fig. S1** Transmission electron microscopy image of cBTO NPs. **Fig. S2** X-ray diffraction spectra of cBTO NPs and partially enlarged spectrum with 2θ range between 44° and 48°. **Fig. S3** X-ray diffraction spectra of tBTO NPs and partially enlarged spectrum with 2θ rangebetween 44° and 48°. **Fig. S4** Scanning electron microscopy images of tBTO NPs treated with H2O2 at 110 °C for 2 h (a) and 125 °C for 1 h (b). **Fig. S5** Hydrodynamic diameter profiles of mtBTO-1, mtBTO-2, and mtBTO-3 NPs. **Fig. S6** The N2 adsorption/desorption isotherms and pore size distribution of mtBTO-1, mtBTO-2, and mtBTO-3 NPs. **Fig. S7** X-ray diffraction spectra of mtBTO-1, mtBTO-2, and mtBTO-3 NPs with magnified 2θ range between 44° and 48°. **Fig. S8** Atomic force microscopy images of tBTO, mtBTO-1, mtBTO-2, and mtBTO-3 NPs. **Fig. S9** HR-TEM characterization of mtBTO-1 NPs. **Fig. S10** HAADF-STEM characterization of mtBTO-1 NPs. **Fig. S11** FTIR spectra of tBTO (blue line) and mtBTO-1 NPs (red line). **Fig. S12** XPS spectra of tBTO and mtBTO-1 NPs and corresponding spectrum for Ti2p and Ba3d. XPS X-ray photoelectron spectroscopy. **Fig. S13** Band structure determination of mtBTO-1 NPs. **Fig. S14** US-initiated degradation kinetics of MB after varied treatments under pH 6.0. **Fig. S15** Temperature curve of 1 ml PBS during ultrasound treatment for 3 min at varied power densities (0.5, 1.0, 1.5, and 2.0 W/cm^2^) and duty ratio (50 and 100%). **Fig. S16** Cell viability of B16 cells after being treated with US at 1.0 W/cm^2^ and 50% duty ratio for a total US time of 1 min at varied US duration parameters. **Fig. S17** CLSM images of B16 cells stained with H2DCF-DA probe after incubation with mtBTO, ZnO2@mtBTO, and SNO-ZnO2@mtBTO NPs with or without ultrasound treatments. **Fig. S18** Statistical analysis of DCF fluorescence intensity of B16 cells after incubation with mtBTO, ZnO2@mtBTO, and SNO-ZnO2@mtBTO NPs with or without ultrasound treatments. **Fig. S19** CLSM images of B16 cells stained with SOSG probe after incubation with mtBTO, ZnO2@mtBTO, and SNOZnO2@ mtBTO NPs with or without ultrasound treatments. **Fig. S20** CLSM images of B16 cells stained with DHE probe after incubation with mtBTO, ZnO2@mtBTO, and SNO-ZnO2@mtBTO NPs with or without ultrasound treatments. **Fig. S21** CLSM images of B16 cells stained with DAX-J2 PON probe after incubation with mtBTO, ZnO2@mtBTO, and SNO-ZnO2@mtBTO NPs with or without ultrasound treatments. **Fig. S22** Statistical analysis of fluorescence intensity of JC-1 in B16 cells after incubation with PBS, mtBTO NPs, ZnO2@mtBTO NPs, and SNO-ZnO2@mtBTO NPs with or without ultrasound treatments. **Fig. S23** Cell viability of RAW264.7 after varied treatments. **Fig. S24** Flow cytometry analysis on RAW264.7 cells after incubating with B16 tumor cells which were pretreated with PBS, ZnO2@mtBTO NPs, SNO-ZnO2@mtBTO NPs, and NZCB NPs (50 μg/ml) with or without US irradiation (1.0 W/cm^2^, 1 min). **Fig. S25** Body weight of mice in each group after treatments of saline, US, mtBTO NPs + US, ZnO2@mtBTO NPs + US, SNOZnO2@ mtBTO NPs + US, and NZCB NPs + US. **Fig. S26** Tumor volume curves of saline, US, mtBTO NPs + US, ZnO2@mtBTO NPs + US, SNOZnO2@ mtBTO NPs + US, and NZCB NPs + US groups during the evaluation timeframe. **Fig. S27** Digital photos of mice from each group on day 12 after treatment of saline, US, mtBTO NPs + US, ZnO2@mtBTO NPs + US, SNO-ZnO2@mtBTO NPs + US, and NZCB NPs + US. **Fig. S28** TUNEL immunofluorescence microscopic images of tumor sections from different groups. **Fig. S29** H&E stained images of major organs of mice including heart, liver, spleen, lung and kidney after treatments of saline, US, mtBTO NPs + US, ZnO2@mtBTO NPs + US, SNO-ZnO2@mtBTO NPs + US, and NZCB NPs + US (Scale bar = 200 μm). **Fig. S30** Biosafety analysis of mice via the complete blood data analysis including white blood cells (WBC), lymphocyte (LYM), granulocytes (GRA), hemoglobin concentration (HGB), mean corpuscular hemoglobin (MCH), red blood cells (RBC), platelet (PLT), albumin (ALB), creatine kinase (CK), creatine kinase-MB (CK-MB), blood creatinine (CREA) and glucose (GLU) markers. **Fig. S31** Biodistribution assay of major organs of mice after intratumoral injection of NZCB NPs (in Ba concentration) at 10 mg/kg for 2, 24, and 48 h. **Fig. S32** Representative gating strategy of spleen lymphocytes for M1-macrophage assay. **Fig. S33** Representative gating strategy of spleen lymphocytes for CD8 T cell assay. **Fig. S34** Statistical analysis of CD86 cells in CD11b F4/80 cells and CD8 T cells in CD3 T cells isolated from spleen tissues of mice after treatments of saline, US, mtBTO NPs + US, ZnO2 mtBTO NPs + US, SNO-ZnO2@mtBTO NPs + US, and NZCB NPs + US. **Fig. S35** Expression of apoptosis-associated genes including BAX, CYCS, CASP3, TNF-α, PRF1 and GZMB between control group and NZCB + US group. **Fig. S36** Relative protein expression of PANoptosis-related proteins after different treatments. **Fig. S37** Western blotting and quantitative analysis of proteins of Fas, FasL, cleaved CASP8, ZBP1, IRF1, and p-STING in different groups.

## Data Availability

The datasets used or analyzed during the current study are available from the corresponding author upon reasonable request.

## Abbreviations

BSA: Bovine serum albumin
BMDCs: Bone marrow dendritic cells
BTO: Barium titanate
cGAMP: Cyclic GMP-AMP synthase
CLSM: Confocal laser scanning microscopy
DAPI: 4′,6-Diamidino-2-phenylindole
DEGs: Differentially expressed genes
DHE: Dihydroethidium
DPBF: 1,3-Diphenylisobenzofuran
ELISA: Enzyme-linked immunosorbent assay
ESR: Electron spin resonance
FITC: Fluorescein isothiocyanate
FTIR: Fourier transform infrared spectra
H_2_O_2_: Hydrogen peroxide
JC-1: 5,5′,6,6′-Tetrachloro-1,1′,3,3′-tetraethylbenzimidazolyl-carbocyanine iodide
KEGG: Kyoto Encyclopedia of Genes and Genomes
MB: Methylene blue
mtBTO: Mesoporous tetragonal BTO
NaOH: Sodium hydroxide
NK: Natural killer
NO: Nitric oxide
NPs: Nanoparticles
NZCB: S-nitrosothiols (SNO)-zinc peroxide (ZnO_2_)@cyclic dinucleotide (CDN)@mtBTO
O_2_: Oxygen
·O_2_^−^: Superoxide anion radical
^1^O_2_: Singlet oxygen
·OH: Hydroxyl radical
ONOO^−^: Peroxynitrite
PBS: Phosphate buffer saline
RNS: Reactive nitrogen species
ROS: Reactive oxygen species
SEM: Scanning electron microscopy
SOSG: Singlet oxygen sensor green
SPDT: Sono-piezodynamic therapy
STING: Stimulator of interferon genes
TEM: Transmission electron microscopy
tBTO: Tetragonal barium titanate
UV–vis: Ultraviolet–visible
US: Ultrasound
XRD: X-ray diffraction
