# Supplementary material for: Ultrasound initiated tumor catalytic PANoptosis by mesoporous piezoelectric nanocatalysts
Source: Mil Med Res. 2025 Jul 30;12:40. doi: 10.1186/s40779-025-00629-9 (PMC12312345; doi:10.1186/s40779-025-00629-9)
Supplement: Supplementary file 1 — Additional file 1. Methods. Fig. S1 Transmission electron microscopy image of cBTO NPs. Fig. S2 X-ray diffraction spectra of cBTO NPs and partially enlarged spectrum with 2θ range between 44° and 48°. Fig. S3 X-ray diffraction spectra of tBTO NPs and partially enlarged spectrum with 2θ rangebetween 44° and 48°. Fig. S4 Scanning electron microscopy images of tBTO NPs treated with H2O2 at 110 °C for 2 h (a) and 125 °C for 1 h (b). Fig. S5 Hydrodynamic diameter profiles of mtBTO-1, mtBTO-2, and mtBTO-3 NPs. Fig. S6 The N2 adsorption/desorption isotherms and pore size distribution of mtBTO-1, mtBTO-2, and mtBTO-3 NPs. Fig. S7 X-ray diffraction spectra of mtBTO-1, mtBTO-2, and mtBTO-3 NPs with magnified 2θ range between 44° and 48°. Fig. S8 Atomic force microscopy images of tBTO, mtBTO-1, mtBTO-2, and mtBTO-3 NPs. Fig. S9 HR-TEM characterization of mtBTO-1 NPs. Fig. S10 HAADF-STEM characterization of mtBTO-1 NPs. Fig. S11 FTIR spectra of tBTO (blue line) and mtBTO-1 NPs (red line). Fig. S12 XPS spectra of tBTO and mtBTO-1 NPs and corresponding spectrum for Ti2p and Ba3d. XPS X-ray photoelectron spectroscopy. Fig. S13 Band structure determination of mtBTO-1 NPs. Fig. S14 US-initiated degradation kinetics of MB after varied treatments under pH 6.0. Fig. S15 Temperature curve of 1 ml PBS during ultrasound treatment for 3 min at varied power densities (0.5, 1.0, 1.5, and 2.0 W/cm2) and duty ratio (50 and 100%). Fig. S16 Cell viability of B16 cells after being treated with US at 1.0 W/cm2 and 50% duty ratio for a total US time of 1 min at varied US duration parameters. Fig. S17 CLSM images of B16 cells stained with H2DCF-DA probe after incubation with mtBTO, ZnO2@mtBTO, and SNO-ZnO2@mtBTO NPs with or without ultrasound treatments. Fig. S18 Statistical analysis of DCF fluorescence intensity of B16 cells after incubation with mtBTO, ZnO2@mtBTO, and SNO-ZnO2@mtBTO NPs with or without ultrasound treatments. Fig. S19 CLSM images of B16 cells stained with SOSG probe after incubatio [file 40779_2025_629_MOESM1_ESM.pdf]

Methods

Materials

Hydrogen peroxide was purchased from Sinopharm Chemical Reagent Co., Ltd. (China). 2-Iminothiolane hydrochloride (Traut's reagent), Zinc chloride, and bovine serum albumin (BSA) purchased from Shanghai Aladdin Biochemical Technology Co. Sodium hydroxide, methanol, and EDTA disodium salt dihydrate were purchased from Shanghai Eon Chemical Technology Co. Tert-butyl nitrite was purchased from Shanghai Titan Chemical Co. Roswell Park Memorial Institute (RPMI) 1640, fetal bovine serum, and phosphate buffer saline (PBS) were purchased from Gibco (USA). 4′,6-diamidino-2-phenylindole (DAPI) was obtained from Beyotime Biotech Inc. Cell Counting Kit-8 (CCK-8), 3,8-diamino-5-[3-(diethylmethylammonio)propyl]-6-phenyl-phenanthridinium diiodide (PI), and 3′,6′-di(O-acetyl)-4′,5′-bis [N,N-bis(carboxymethyl) aminomethyl] fluorescein-tetraacetoxymethyl ester (Calcein-AM) were purchased from Dojindo Molecular Technologies. 5,5′,6,6′-Tetrachloro-1,1′,3,3′-tetraethylimida-carbocyanine iodide (JC-1) was purchased from Beyotime Biotechnology. Hydrogen Peroxide Assay Kit was purchased from Beyotime Biotechnology. Dihydroethidium (DHE) was purchased from QiYue Bio, China. 2',7'-dichlorodihydrofluorescein diacetate (H_2DCF-DA), Diaminofluorescein-FM diacetate (DAF-FM DA), 2',3'-cGAMP sodium was purchased from MedChemExpress. The Fluorimetric Intracellular Peroxynitrite Assay Kit was purchased from the United States AAT Bioquest. Poly (N-iso-propylacrylamide) was purchased from United States Polysciences Companies. CD45-APC-cy7 (557659), CD3-BUV395 (563565), CD4-BV605 (563151), CD8-BV786 (563332), CD11b-FITC (557396), F4/80-BV421 (565411), CD86-APC (558703), and CD206-PE (568273) were purchased from BD Biosciences. cGAMP ELISA kit, Western blotting antibodies including nucleotide oligomerization domain-like receptor pyrin domain

containing 3 (NLRP3), granzyme B/H, tumor necrosis factor-α (TNF-α), mixed lineage kinase domain-like protein (MLKL), cytochrome c, cleaved caspase 3 (CASP3), total and cleaved N-terminal GSDMD, B-cell lymphoma 2 associated X (BAX), receptor-interacting serine/threonine-protein kinase 1 (RIPK1), total and cleaved CASP1, and PRF1 were purchased from Abimat Medical Technology Co., Ltd. Interleukin-1β (IL-1β), TNF-α and interferon-β (IFN-β) ELISA kit were purchased from Lianshuo Biological Technology Co., Ltd.

Instrument and characterization

Scanning electron microscopy (SEM) images were recorded on a field-emission Magellan 400 microscope (FEI Company, US). X-ray diffraction (XRD) was measured on a Rigaku D/MAX-2200 PC XRD system (Cu Kα, λ = 1.54 Å, 40 mA, and 40 kV). Ultraviolet-visible-near-infrared spectrophotometer (UV-vis-NIR) absorption spectra were performed on UV-3600 Shimadzu UV-vis-NIR spectrometer. The piezoelectric properties were tested with Bruker Dimension Icon AFM instrument. Fourier transform infrared spectroscopy (FTIR) spectra were obtained on Nicolet iS 10, Thermo Scientific, scanning in the range of 200 − 1000/cm. ESR characterization was performed on JEOL-FA200, JEOL, Japan.

In vitro cytotoxicity assay

B16 cells were inoculated at 1×10^4 cells/well in 96-well plates for 24 h until the cells were attached. Then, the medium was removed and 1640 medium containing mesoporous tetragonal barium titanate (mtBTO) nanoparticles (NPs), zinc peroxide (ZnO_2)@mtBTO NPs, and S-nitrosothiols (SNO)-ZnO_2@mtBTO NPs at varied concentrations (0, 25, 50, 100, 200 and 400 μg/ml) were added, with or without further ultrasound (US) irradiation (0, 0.5, 0.75, 1.0, 1.5 W/cm^2, 1 min) after 4 h. CCK-8 assay was carried out 6 h post-dosing.

Cell staining (live/dead, JC-1) and confocal observations

B16 cells were inoculated at 1 × 10^5 cells/well in 12-well plates for 12 h, followed by the addition of mtBTO NPs, ZnO_2@mtBTO NPs, and SNO-ZnO_2@mtBTO NPs at 60 μg/ml, with or without US irradiation (1.0 W/cm^2, 1 min) after 4 h post-dosing.

To visualize the distribution of the live/dead cells, cells were stained with Calcein-AM (10 μmol/L, $\lambda_{Ex}/\lambda_{Em}$ = 490 nm/515 nm) and PI probes (2 μmol/L, $\lambda_{Ex}/\lambda_{Em}$ = 530 nm/580 nm) for fluorescence observation at 6 h post-dosing.

To evaluate the mitochondrial membrane potential changes, JC-1 dyes were incubated to the cells at 6 h post-dosing. JC-1 monomers with green fluorescence (E_x/E_m = 510 nm/527 nm) were formed since membrane potential decreases, indicating potential cell apoptosis.

Cell staining [singlet oxygen ($^1O_2$), superoxide anion radicals ($\cdot O_2^-$), nitric oxide (NO), and peroxynitrite ($ONOO^-$)] and confocal observations

B16 cells were inoculated at 1 × 10^5 cells/well in 12-well plates for 24 h. mtBTO NPs, ZnO_2@mtBTO NPs, and SNO-ZnO_2@mtBTO NPs at 50 μg/ml were co-incubated for 4 h, followed by or without US irradiation (1.0 W/cm^2, 1 min, 50% duty ratio). After US treatment, cells were stained with varied fluorescent probes to evaluate radical production, including H_2DCF-DA (100 μmol/L, 200 μl) for total reactive oxygen species (ROS) evaluation, 4-amino-5-methylamino-2,7-difluorofluorescein diacetate (DA) (50 μmol/L) for intracellular NO production, DHE for $\cdot O_2^-$ detection and DAX-J2™ PON Green (AAT Bioquest, 10 μmol/L) for intracellular NO evaluation. Fluorescent probe staining protocols were carried out according to instructions.

Western blotting analysis

For immunoblotting of proteins, B16 cells were inoculated with mtBTO, ZnO_2@mtBTO, SNO-

ZnO_2@mtBTO NPs (50 μg/ml) overnight, followed by US treatment and further co-incubation for 4 h. Afterward, cells were washed with cold PBS 3 times and treated with trypsin. Cells were then collected and treated with RIPA-containing protease inhibitor, followed by centrifugation at 12,000 rpm for 15 min for protein collection. Loading buffer was added into supernatants and heated at 100 °C for 10 min and further stored at –20 °C. Proteins of B16 tumor tissues were prepared using 50 mg tissues per sample for homogenization at 4 °C using beads.

US experiment setup

During in vitro experiments, US parameters were set as follows: power density 1.0 W/cm^2, 50% duty ratio, 1 MHz, and 1 min. US was conducted for 1 min to avoid cell detachment. During in vivo experiments, the irradiation time was set to 3 min with other US parameters unchanged. The duty ratio was set as 50% to avoid the innate US cavitation effect.

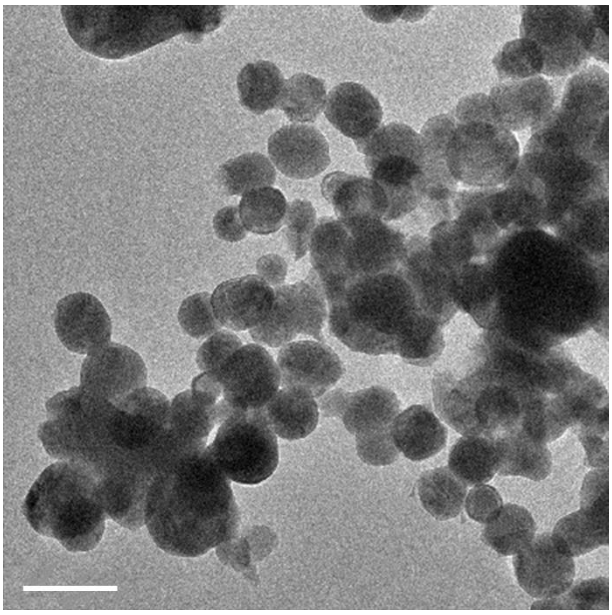

Fig. S1 Transmission electron microscopy image of cubic BTO NPs. Scale bar = 200 nm. BTO barium titanate, NPs nanoparticles

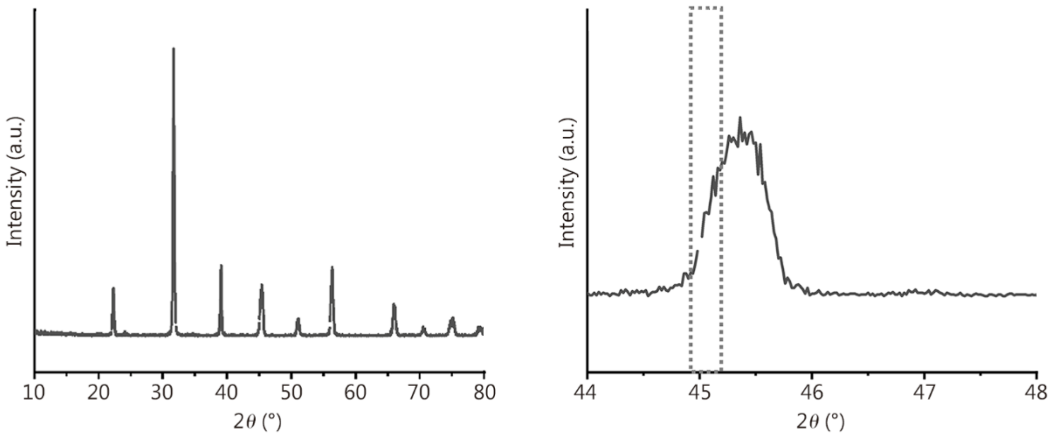

Fig. S2 X-ray diffraction spectra of cubic BTO NPs and partially enlarged spectrum with 2θ range between 44 − 48°. BTO barium titanate, NPs nanoparticles

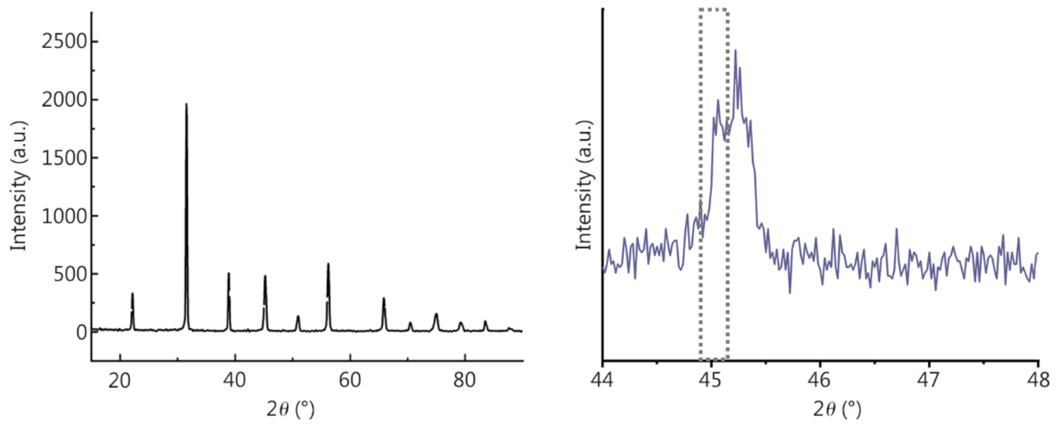

Fig. S3 X-ray diffraction spectra of tBTO NPs and partially enlarged spectrum with 2θ range between 44 − 48°. tBTO tetragonal barium titanate, NPs nanoparticles

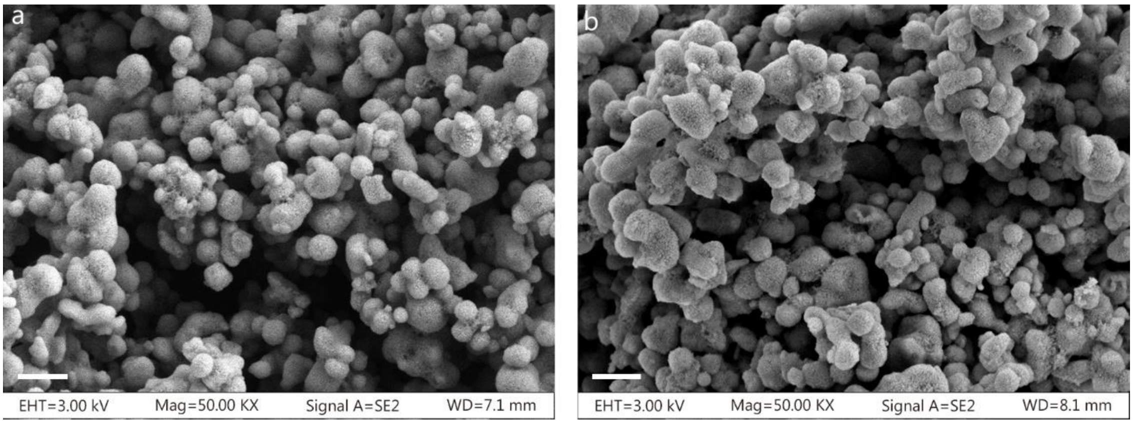

Fig. S4 Scanning electron microscopy images of tBTO NPs treated with H_2O_2 at 110 °C for 2 h (**a**) and 125 °C for 1 h (**b**). Scale bar = 200 nm. tBTO tetragonal barium titanate, NPs nanoparticles

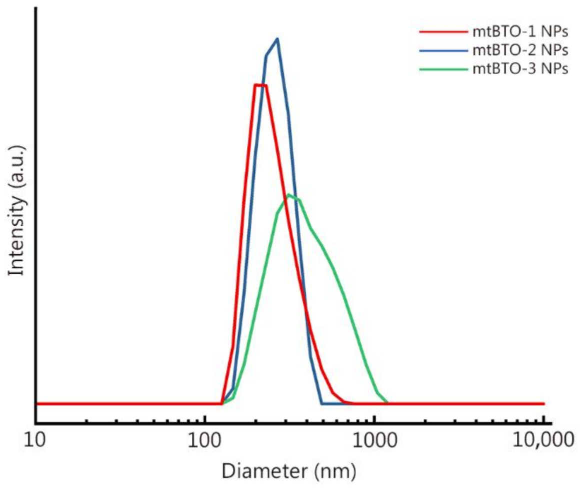

Fig. S5 Hydrodynamic diameter profiles of mtBTO-1, mtBTO-2, and mtBTO-3 NPs. mtBTO mesoporous tetragonal barium titanate, NPs nanoparticles

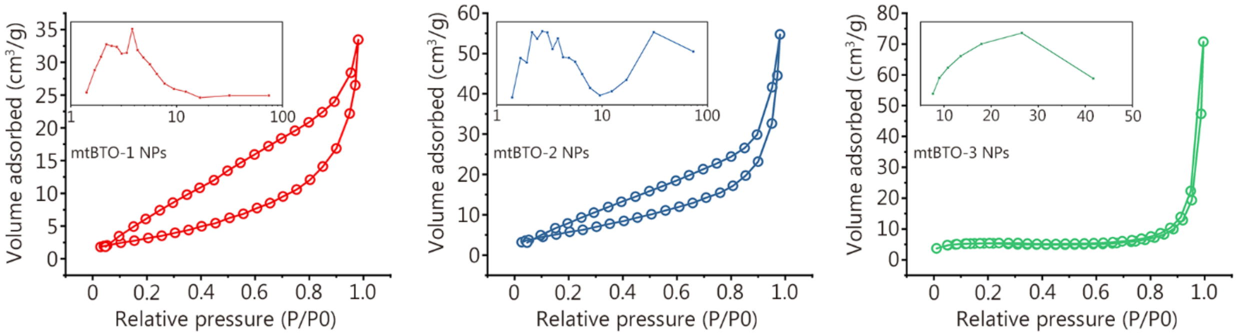

Fig. S6 The N_2 adsorption/desorption isotherms and pore size distribution of mtBTO-1, mtBTO-2, and mtBTO-3 NPs. mtBTO mesoporous tetragonal barium titanate, NPs nanoparticles

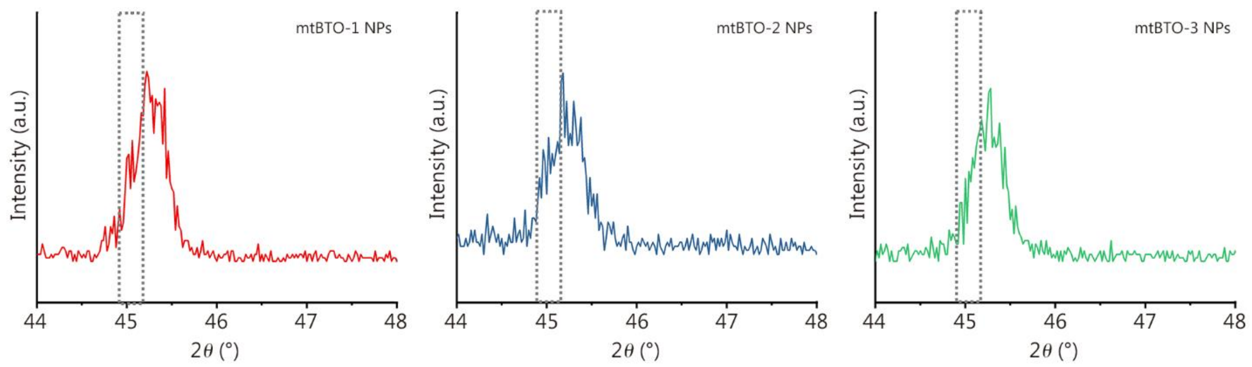

Fig. S7 X-ray diffraction spectra of mtBTO-1, mtBTO-2, and mtBTO-3 NPs with magnified 2θ range between 44 − 48°. mtBTO mesoporous tetragonal barium titanate, NPs nanoparticles

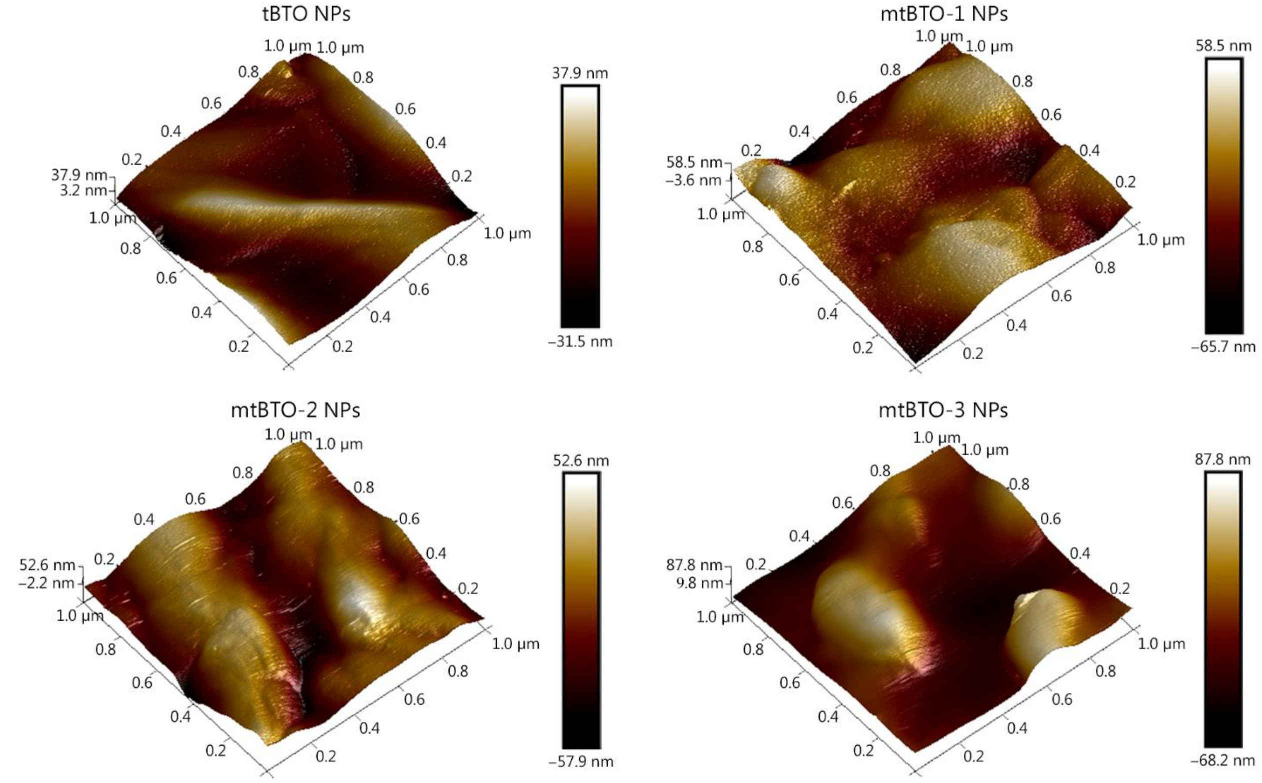

Fig. S8 Atomic force microscopy images of tBTO, mtBTO-1, mtBTO-2, and mtBTO-3 NPs. tBTO tetragonal barium titanate, mtBTO mesoporous tetragonal barium titanate, NPs nanoparticles

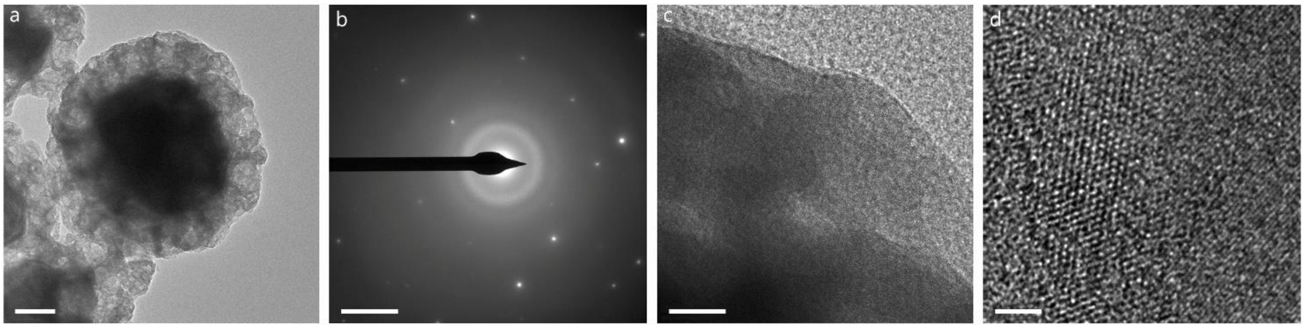

Fig. S9 HR-TEM characterization of mtBTO-1 NPs. TEM image (**a**, scale bar = 50 nm), selected-area electron diffraction (**b**, scale bar = 5 nm), lattice fringes of mtBTO-1 (**c** and **d**, scale bars = 10 nm and 2 nm) from HR-TEM. HR-TEM high-resolution transmission electron microscopy, mtBTO mesoporous tetragonal barium titanate, NPs nanoparticles

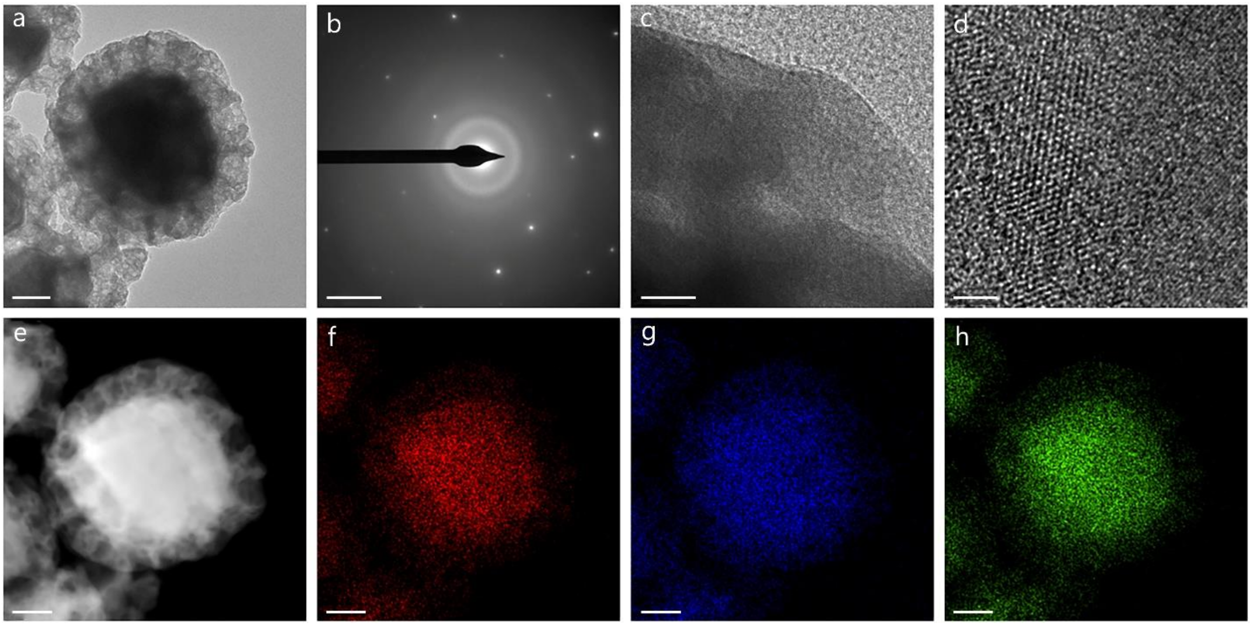

Fig. S10 HAADF-STEM characterization of mtBTO-1 NPs. HAADF-STEM image (**a** and **e**) and corresponding TEM elemental mapping of Ba (**b** and **f**), O (**c** and **g**), Ti (**d** and **h**) of mtBTO-1 NPs at low magnification (**a-d**) and high magnification of a representative particle (**e-h**). Scale bar = 50 nm (**a**, **e-h**), scale bar = 5 nm (**b**), scale bar = 10 nm (**c**), scale bar = 2 nm (**e**). HAADF-STEM high-angle annular dark-field scanning transmission electron microscopy. TEM transmission electron microscopy, mtBTO mesoporous tetragonal barium titanate, NPs nanoparticles

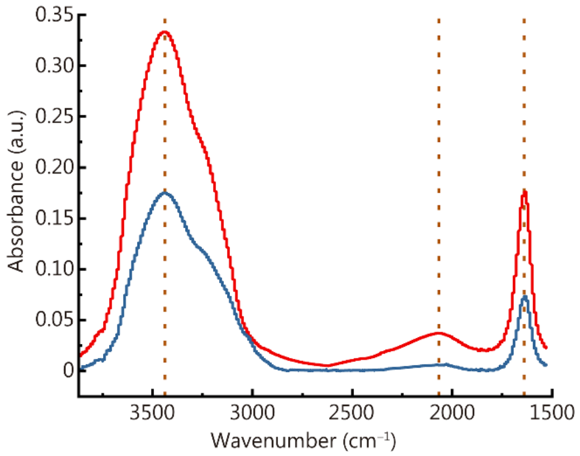

Fig. S11 FTIR spectra of tBTO (blue line) and mtBTO-1 NPs (red line). FTIR Fourier transform infrared spectroscopy, tBTO tetragonal barium titanate, mtBTO mesoporous tetragonal barium titanate, NPs nanoparticles

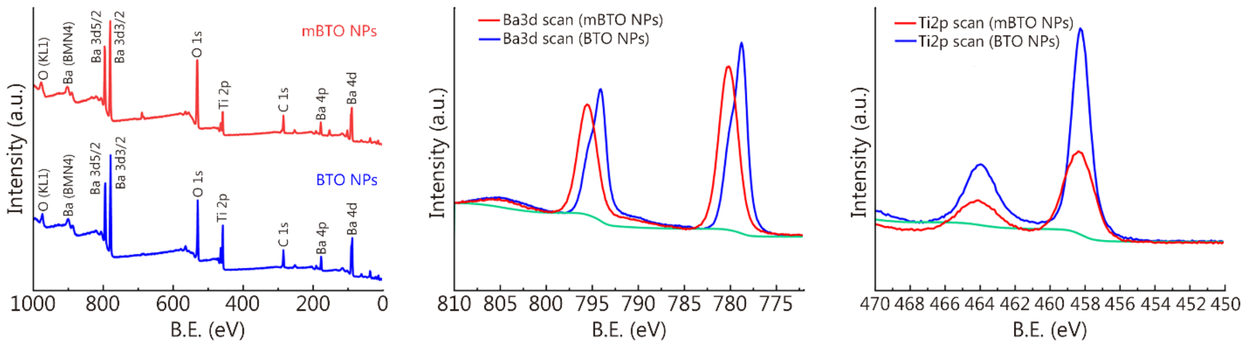

Fig. S12 XPS spectra of tBTO and mtBTO-1 NPs and corresponding spectrum for Ba3d and Ti2p. XPS X-ray photoelectron spectroscopy, tBTO tetragonal barium titanate, mtBTO mesoporous tetragonal barium titanate, NPs nanoparticles

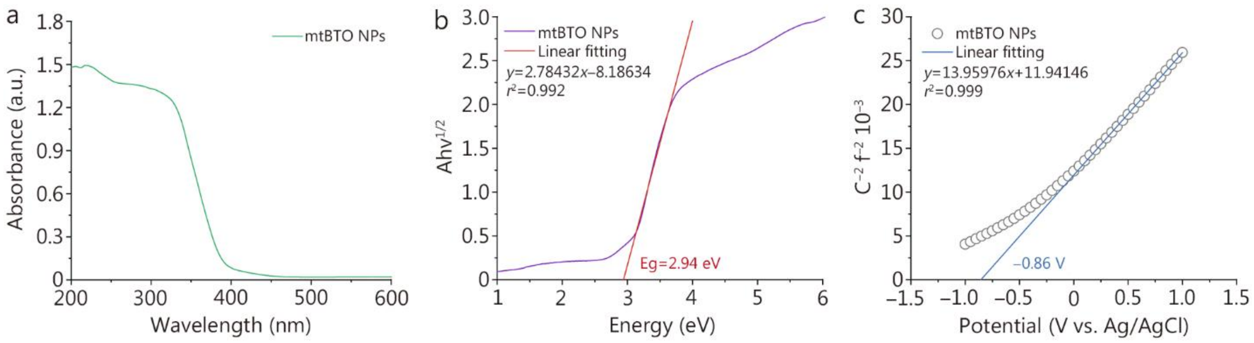

Fig. S13 Band structure determination of mtBTO-1 NPs. **a** Diffuse reflection UV-vis spectrum of mtBTO-1 NPs. **b** Tauc plot of mtBTO-1 NPs for band gap determination. **c** Mott-Schottky curve of mtBTO NPs. mtBTO mesoporous tetragonal barium titanate, NPs nanoparticles

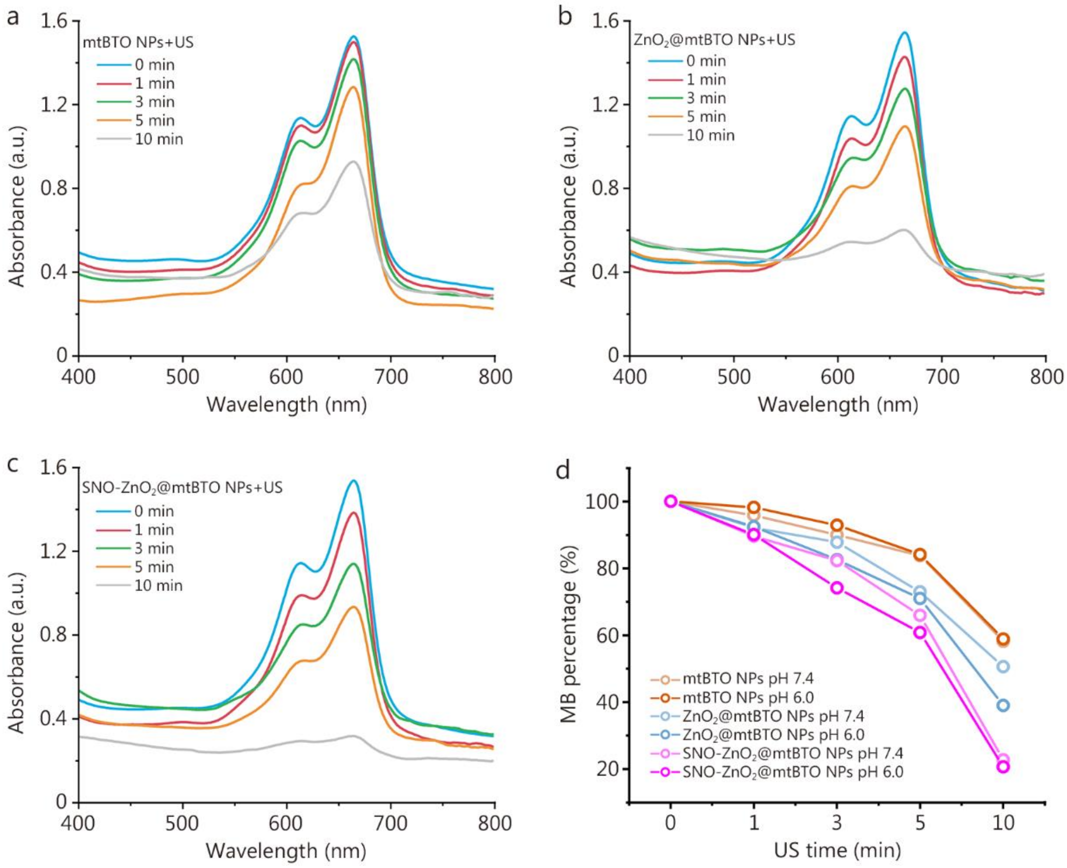

Fig. S14 US-initiated degradation kinetics of MB after varied treatments under pH 6.0. Optical absorbance curves of MB after co-incubation with mtBTO (**a**), ZnO_2@mtBTO (**b**), and SNO-ZnO_2@mtBTO NPs (**c**) at 20 µg/ml after US irradiation (1.0 W/cm^2) for 1, 3, 5 and 10 min under pH 6.0. **d** Corresponding statistical analysis of MB bleach percentage at 664 nm of these groups. MB methylene blue, US ultrasound, SNO-ZnO_2@mtBTO S-nitrosothiols (SNO)-zinc peroxide (ZnO_2)@mesoporous tetragonal barium titanate, NPs nanoparticles

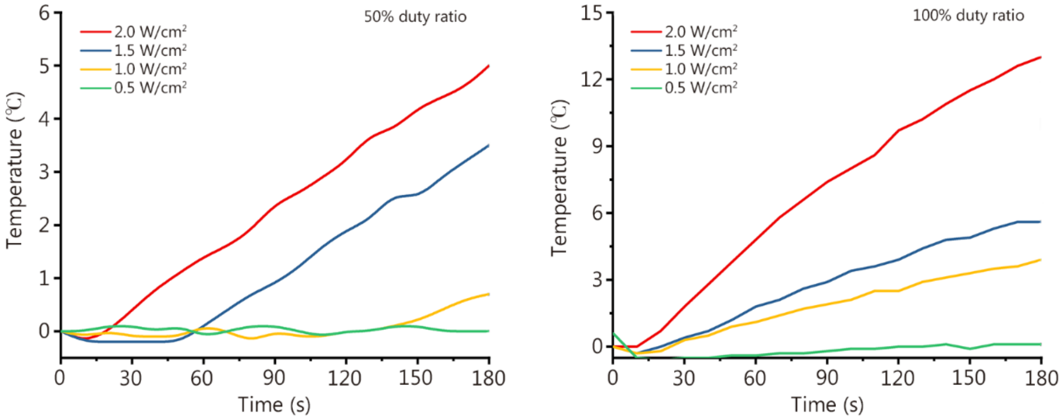

Fig. S15 Temperature curve of 1 ml PBS during ultrasound treatment for 3 min at varied power densities (0.5, 1.0, 1.5, and 2.0 W/cm^2) and duty ratio (50 and 100%)

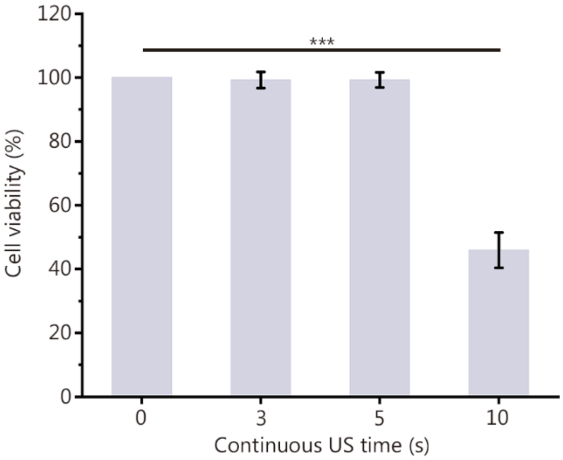

Fig. S16 Cell viability of B16 cells after being treated with US at 1.0 W/cm^2 and 50% duty ratio for a total US time of 1 min at varied US duration parameters. $^{***}P < 0.001$. US ultrasound

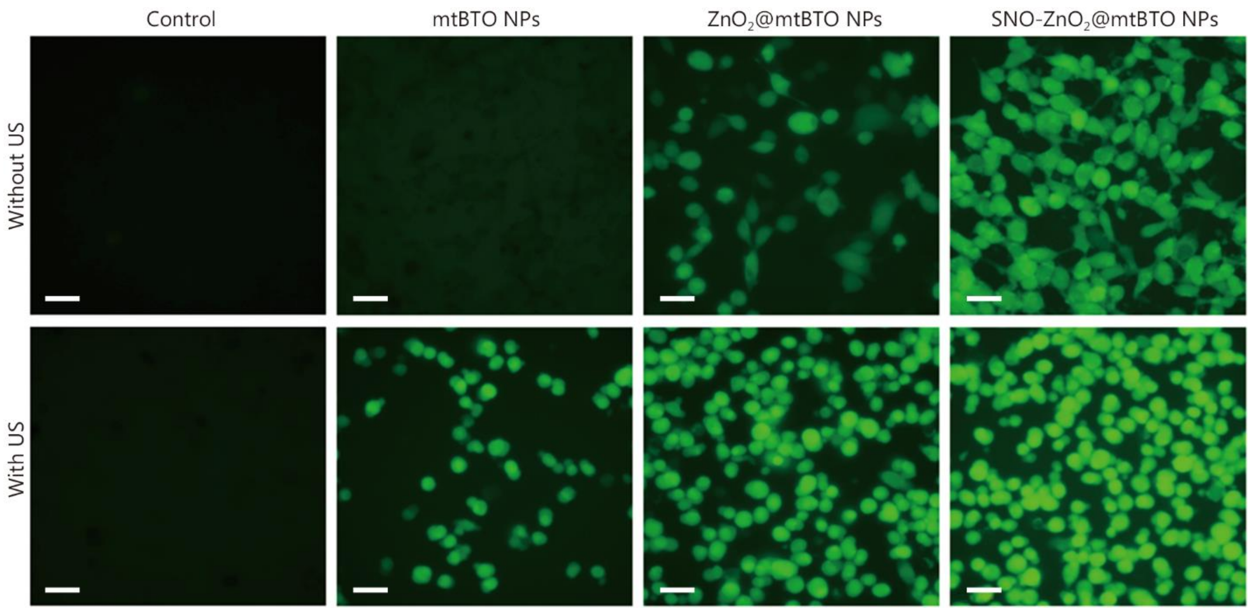

Fig. S17 CLSM images of B16 cells stained with H_2DCF-DA probe after incubation with mtBTO, ZnO_2@mtBTO, and SNO-ZnO_2@mtBTO NPs with or without ultrasound (US) treatments. Scale bar = 20 μm. CLSM confocal laser scanning microscopy, H_2DCF-DA 2,7-dichlorodihydrofluorescein diacetate, SNO-ZnO_2@mtBTO S-nitrosothiols (SNO)-zinc peroxide (ZnO_2)@mesoporous tetragonal barium titanate, NPs nanoparticles

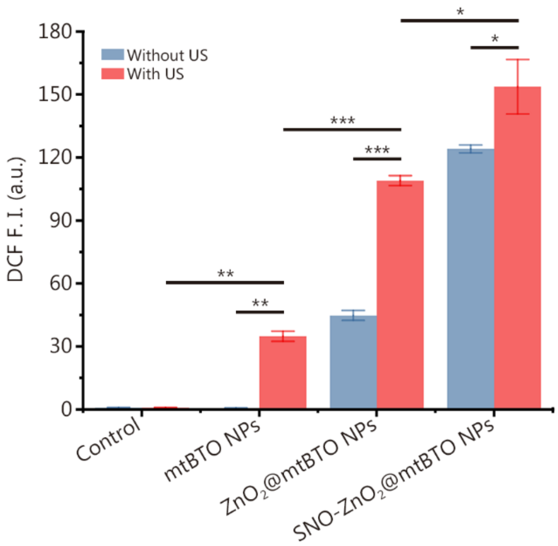

Fig. S18 Statistical analysis of DCF fluorescence intensity of B16 cells after incubation with mtBTO, ZnO_2@mtBTO, and SNO-ZnO_2@mtBTO NPs with or without ultrasound (US) treatment. $^{*}P < 0.05$, $^{**}P < 0.01$, $^{***}P < 0.001$. SNO-ZnO_2@mtBTO S-nitrosothiols (SNO)-zinc peroxide (ZnO_2)@mesoporous tetragonal barium titanate, NPs nanoparticles, DCF 2',7'-dichlorofluorescein

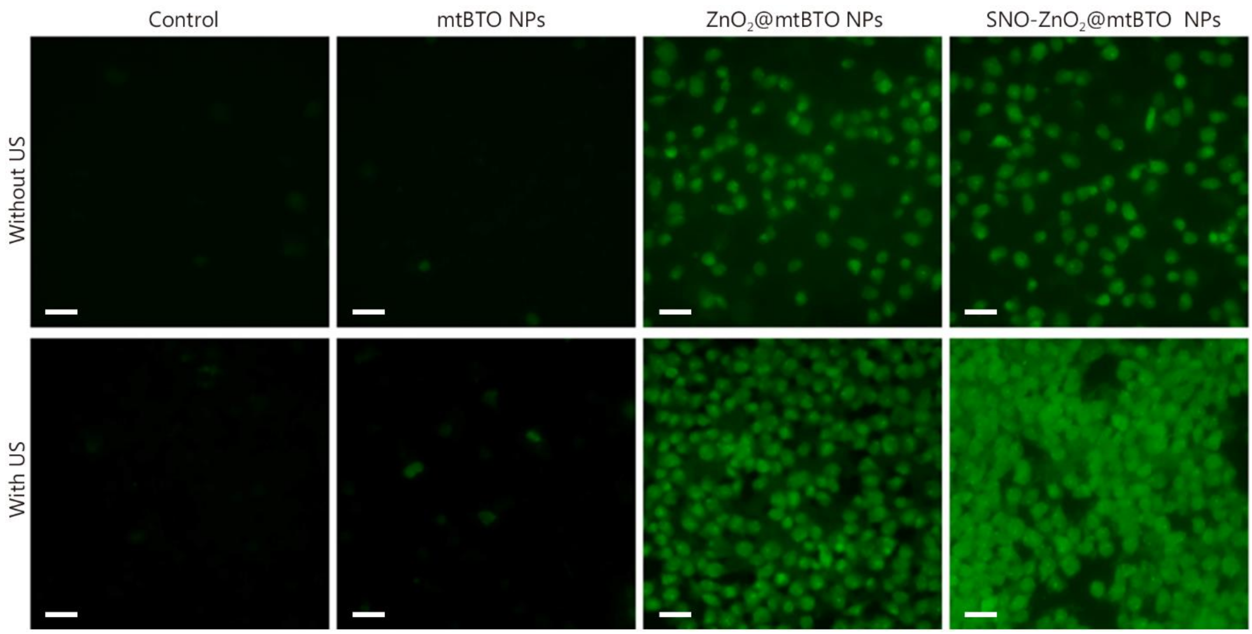

Fig. S19 CLSM images of B16 cells stained with SOSG probe after incubation with mtBTO, ZnO_2@mtBTO, and SNO-ZnO_2@mtBTO NPs with or without ultrasound (US) treatments. Scale bar = 20 μm. SOSG singlet oxygen sensor green, CLSM confocal laser scanning microscopy, SNO-ZnO_2@mtBTO S-nitrosothiols (SNO)-zinc peroxide (ZnO_2)@mesoporous tetragonal barium titanate, NPs nanoparticles

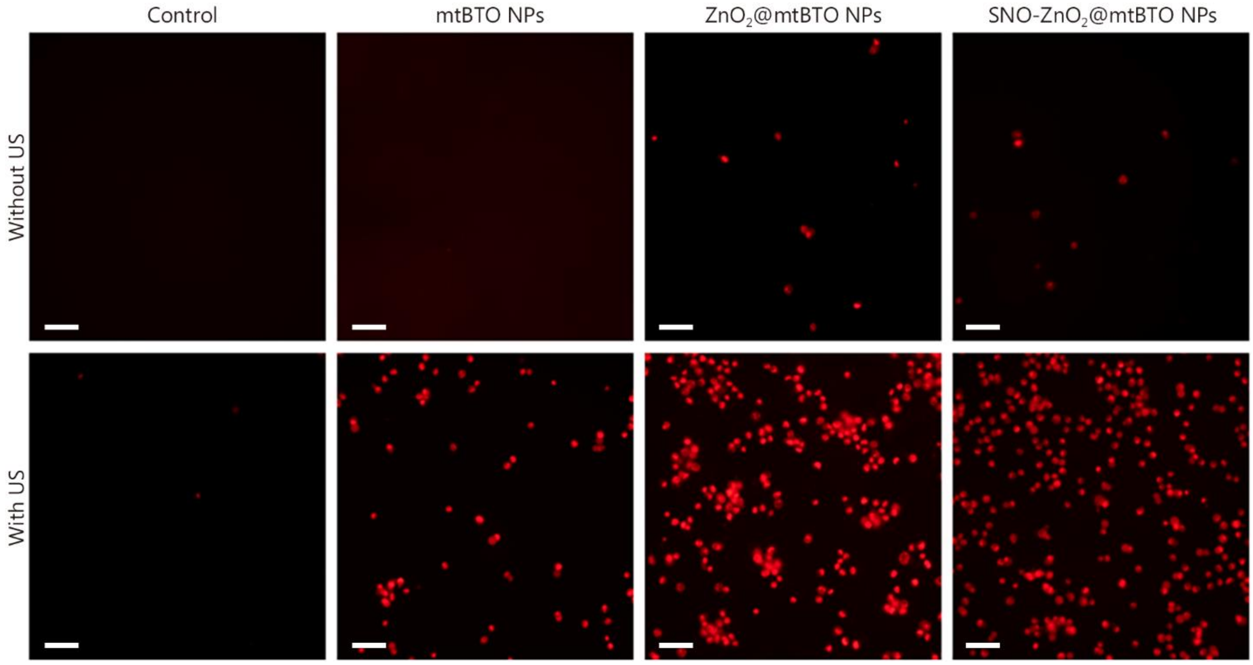

Fig. S20 CLSM images of B16 cells stained with DHE probe after incubation with mtBTO, ZnO_2@mtBTO, and SNO-ZnO_2@mtBTO NPs with or without ultrasound (US) treatments. Scale bar = 50 μm. DHE dihydroethidium, CLSM confocal laser scanning microscopy, SNO-ZnO_2@mtBTO S-nitrosothiols (SNO)-zinc peroxide (ZnO_2)@mesoporous tetragonal barium titanate, NPs nanoparticles

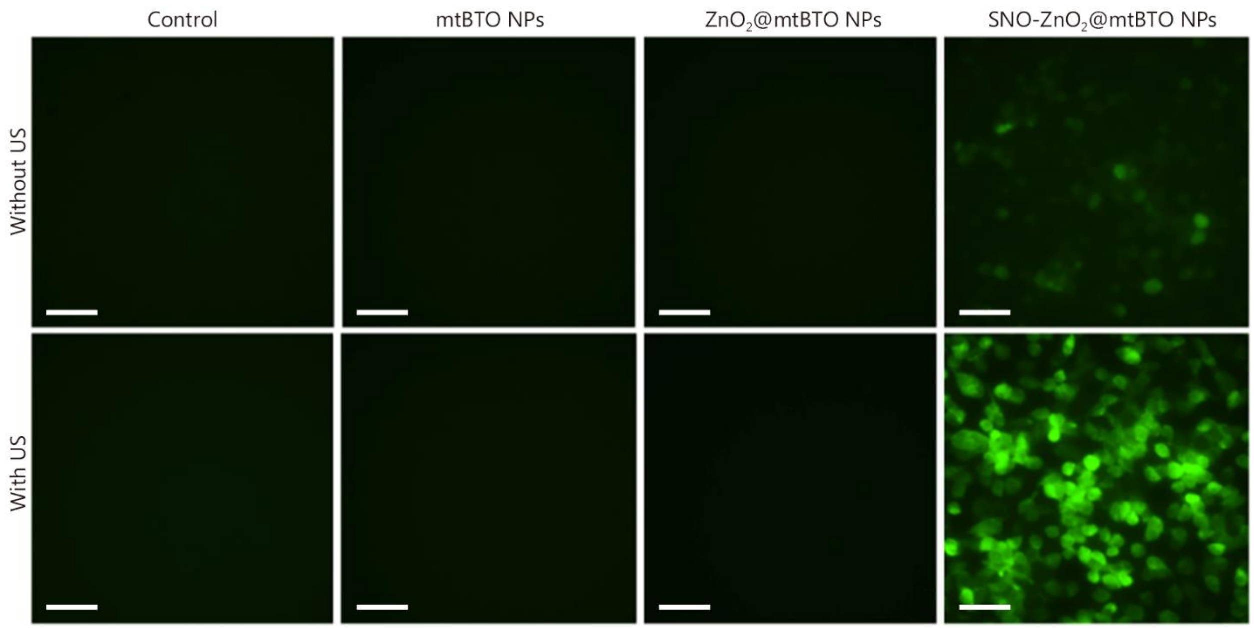

Fig. S21 CLSM images of B16 cells stained with DAX-J2 PON probe after incubation with mtBTO, ZnO_2@mtBTO, and SNO-ZnO_2@mtBTO NPs with or without ultrasound (US) treatments. Scale bar = 20 μm. CLSM confocal laser scanning microscopy, SNO-ZnO_2@mtBTO S-nitrosothiols (SNO)-zinc peroxide (ZnO_2)@mesoporous tetragonal barium titanate, NPs nanoparticles

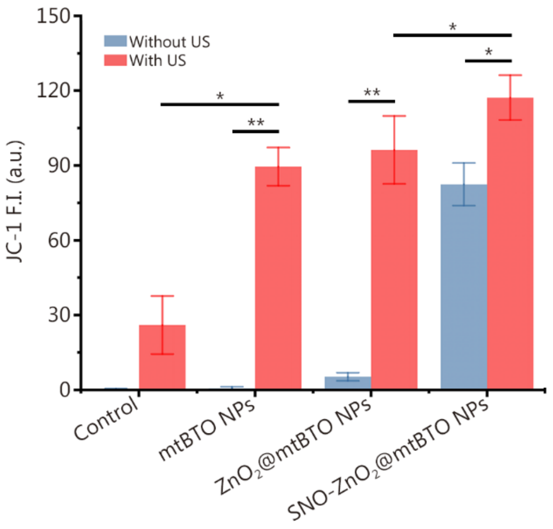

Fig. S22 Statistical analysis of fluorescence intensity of JC-1 in B16 cells after incubation with PBS, mtBTO NPs, ZnO_2@mtBTO NPs, and SNO-ZnO_2@mtBTO NPs with or without ultrasound (US) treatments. $^{*}P < 0.05$, $^{**}P < 0.01$. JC-1 5,5’,6,6’-tetrachloro-1,1’,3,3’-tetraethylbenzimidazolyl-carbocyanine iodide, SNO-ZnO_2@mtBTO S-nitrosothiols (SNO)-zinc peroxide (ZnO_2)@mesoporous tetragonal barium titanate, NPs nanoparticles

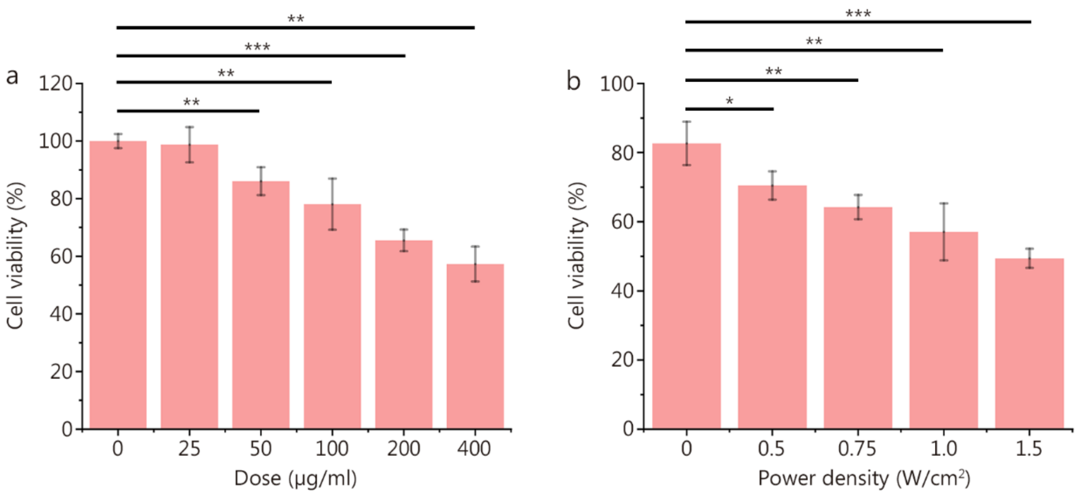

Fig. S23 Cell viability of RAW264.7 after varied treatments. **a** Cell viability of NZCB NPs on RAW264.7 at varied concentrations (0, 25, 50, 100, 200 and 400 μg/ml). **b** Cell viability of NZCB NPs at 50 μg/ml on RAW264.7 in the presence of US at varied power densities (0, 0.5, 0.75, 1.0 and 1.5 W/cm^2). $^{*}P < 0.05$, $^{**}P < 0.01$, $^{***}P < 0.001$. NZCB S-nitrosothiols (SNO)-zinc peroxide (ZnO_2)@cyclic dinucleotide (CDN)@mesoporous tetragonal barium titanate (mtBTO), NPs nanoparticles, US ultrasound

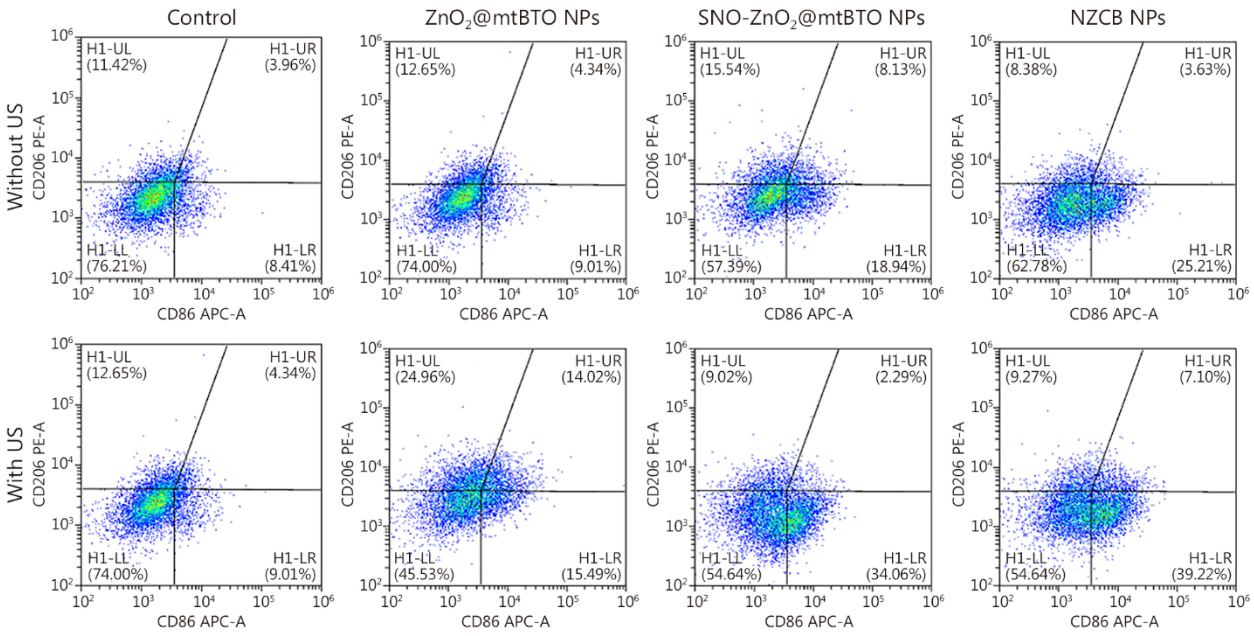

Fig. S24 Flow cytometry analysis on RAW264.7 cells after incubating with B16 tumor cells which were pretreated with PBS, ZnO_2@mtBTO NPs, SNO-ZnO_2@mtBTO NPs, and NZCB NPs (50 µg/ml) with or without US irradiation (1.0 W/cm^2, 1 min). NZCB S-nitrosothiols (SNO)-zinc peroxide (ZnO_2)@cyclic dinucleotide (CDN)@mesoporous tetragonal barium titanate (mtBTO), NPs nanoparticles, US ultrasound

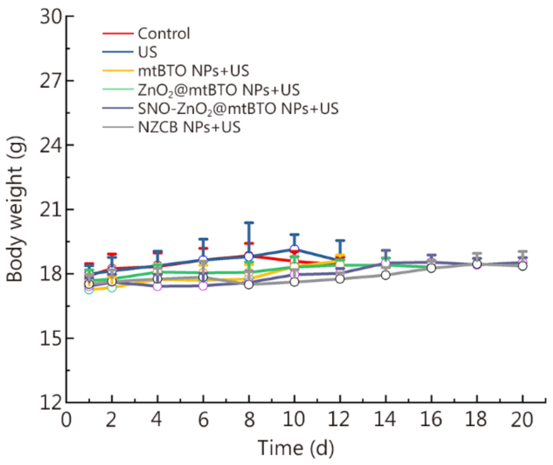

Fig. S25 Body weight of mice in each group after treatments of saline, US, mtBTO NPs + US, ZnO_2@mtBTO NPs +US, SNO-ZnO_2@mtBTO NPs + US, and NZCB NPs + US. NZCB S-nitrosothiols (SNO)-zinc peroxide (ZnO_2)@cyclic dinucleotide (CDN)@mesoporous tetragonal barium titanate (mtBTO), US ultrasound, NPs nanoparticles

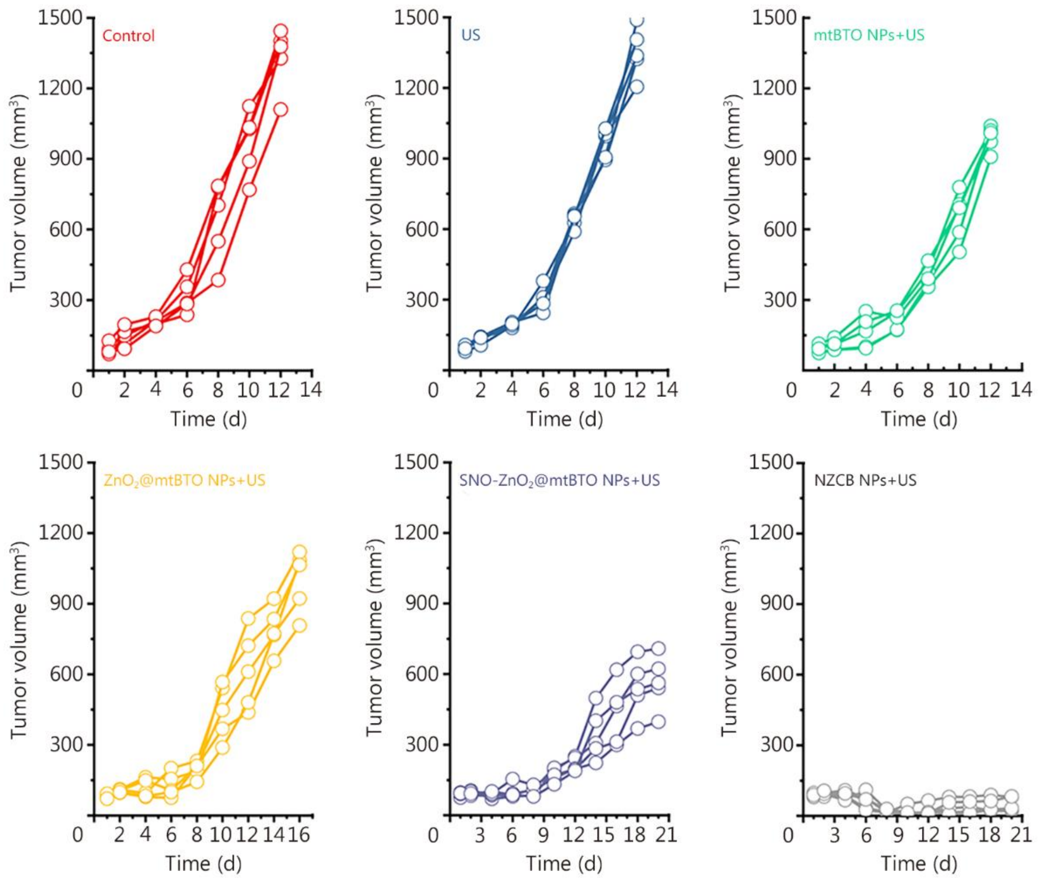

Fig. S26 Tumor volume curves of saline, US, mtBTO NPs + US, ZnO_2@mtBTO NPs +US, SNO-ZnO_2@mtBTO NPs + US, and NZCB NPs + US groups during the evaluation timeframe. NZCB S-nitrosothiols (SNO)-zinc peroxide (ZnO_2)@cyclic dinucleotide (CDN)@mesoporous tetragonal barium titanate (mtBTO), US ultrasound, NPs nanoparticles

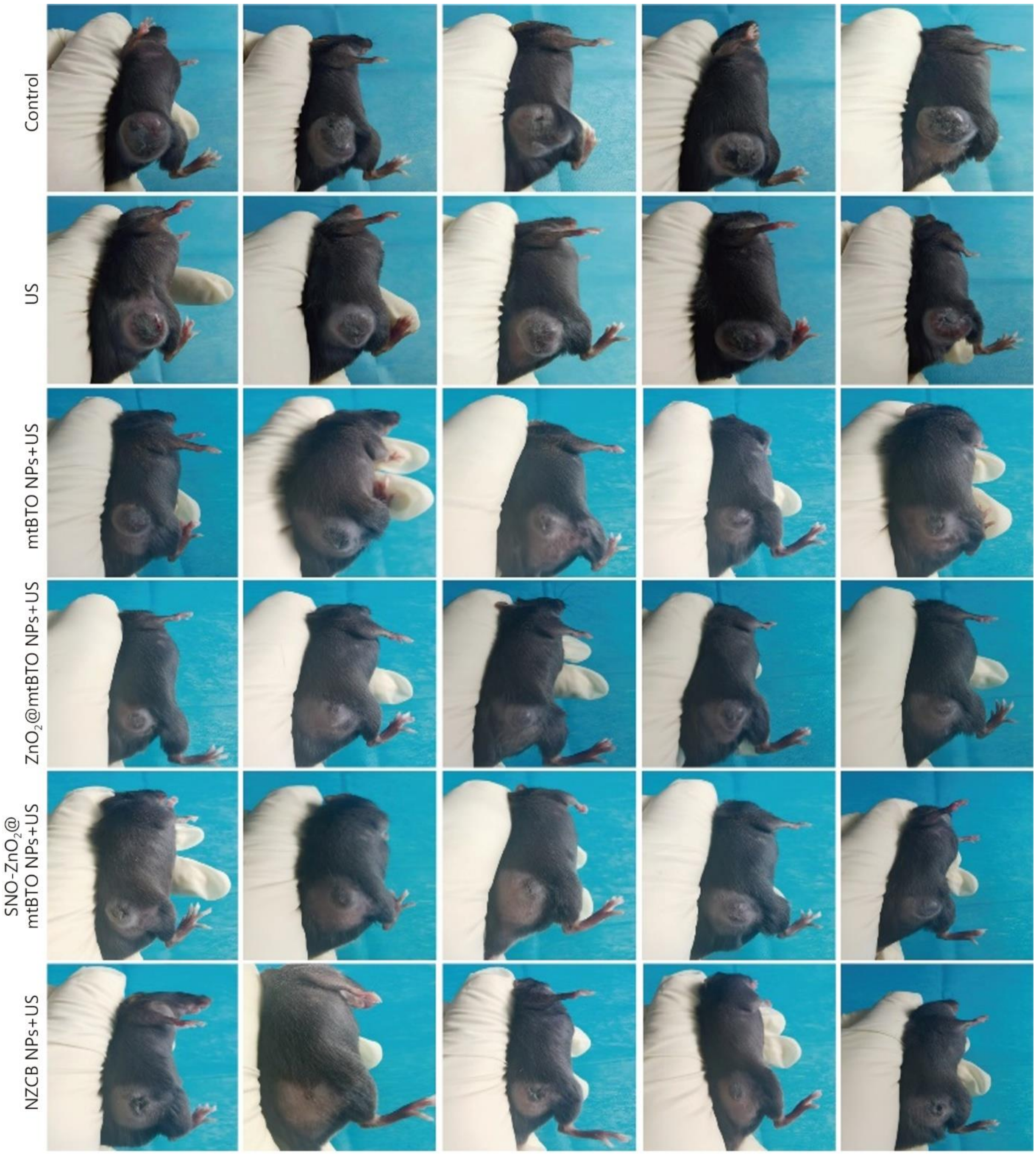

Fig. S27 Digital photos of mice from each group on day 12 after treatment of saline, US, mtBTO NPs + US, ZnO_2@mtBTO NPs +US, SNO-ZnO_2@mtBTO NPs + US, and NZCB NPs + US. NZCB S-nitrosothiols (SNO)-zinc peroxide (ZnO_2)@cyclic dinucleotide (CDN)@mesoporous tetragonal barium titanate (mtBTO), US ultrasound, NPs nanoparticles

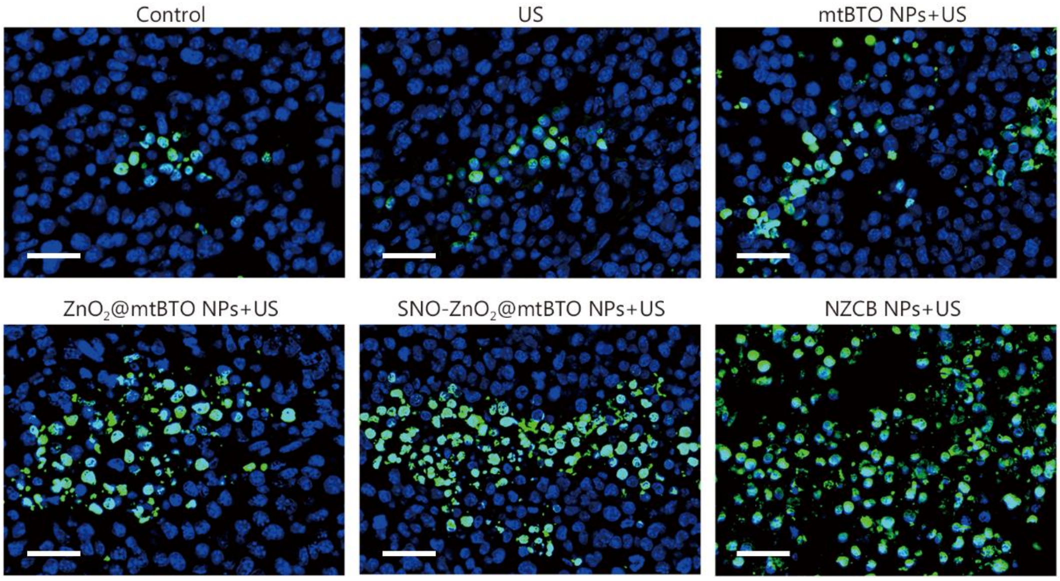

Fig. S28 TUNEL immunofluorescence microscopic images of tumor sections from different groups. Scale bar = 20 μm. TUNEL terminal deoxynucleotidyl transferase dUTP nick end labeling, NZCB S-nitrosothiols (SNO)-zinc peroxide (ZnO_2)@cyclic dinucleotide (CDN)@mesoporous tetragonal barium titanate (mtBTO), US ultrasound, NPs nanoparticles

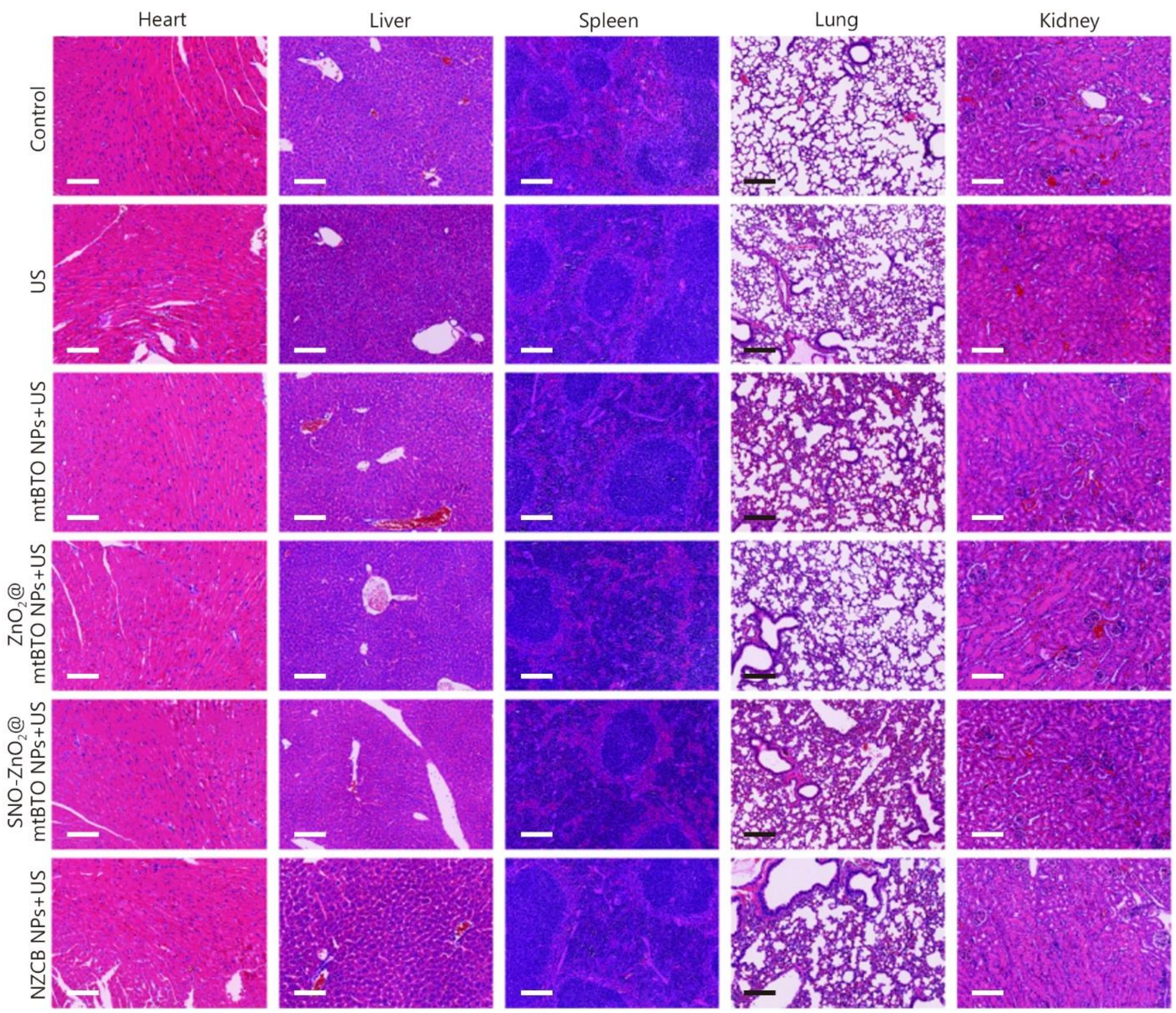

Fig. S29 H&E stained images of major organs of mice including heart, liver, spleen, lung and kidney after treatments of saline, US, mtBTO NPs + US, ZnO_2@mtBTO NPs + US, SNO-ZnO_2@mtBTO NPs + US, and NZCB NPs + US. Scale bar = 200 μm. H&E hematoxylin and eosin, NZCB S-nitrosothiols (SNO)-zinc peroxide (ZnO_2)@cyclic dinucleotide (CDN)@mesoporous tetragonal barium titanate (mtBTO), US ultrasound, NPs nanoparticles

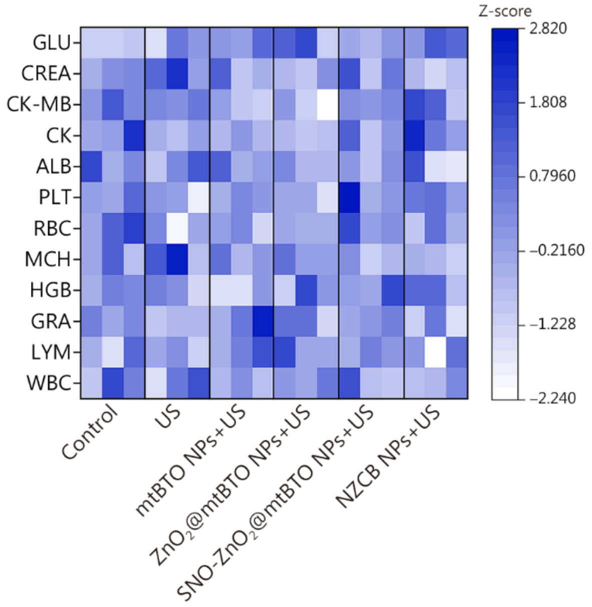

Fig. S30 Biosafety analysis of mice via the complete blood data analysis including white blood cells (WBC), lymphocyte (LYM), granulocytes (GRA), hemoglobin concentration (HGB), mean corpuscular hemoglobin (MCH), red blood cells (RBC), platelet (PLT), albumin (ALB), creatine kinase (CK), creatine kinase-MB (CK-MB), blood creatinine (CREA) and glucose (GLU) markers. NZCB S-nitrosothiols (SNO)-zinc peroxide (ZnO_2)@cyclic dinucleotide (CDN)@mesoporous tetragonal barium titanate (mtBTO), US ultrasound, NPs nanoparticles

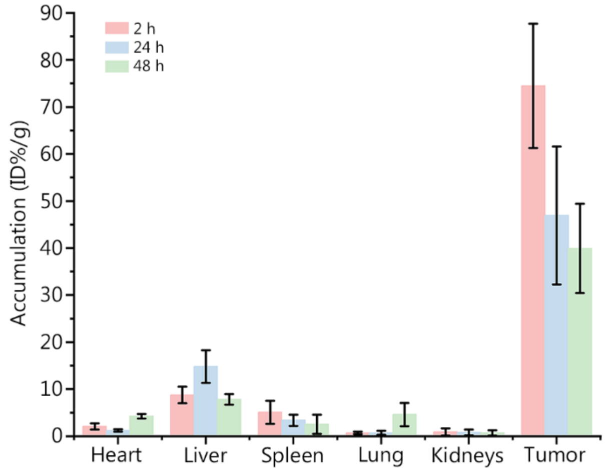

Fig. S31 Biodistribution assay of major organs of mice after intratumoral injection of NZCB NPs (in Ba concentration) at 10 mg/kg for 2, 24, and 48 h. NZCB NPs S-nitrosothiols (SNO)-zinc peroxide (ZnO_2)@cyclic dinucleotide (CDN)@mesoporous tetragonal barium titanate (mtBTO) nanoparticles, US ultrasound

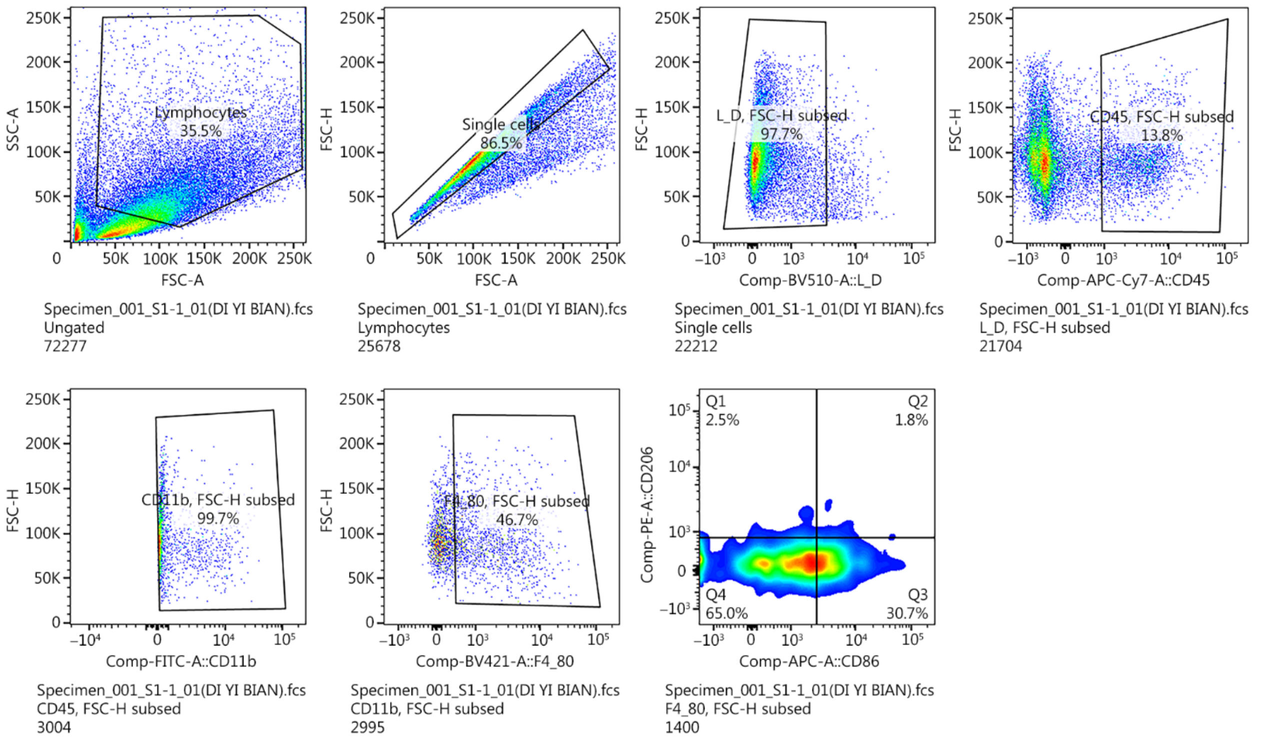

Fig. S32 Representative gating strategy of spleen lymphocytes for M1-macrophage assay

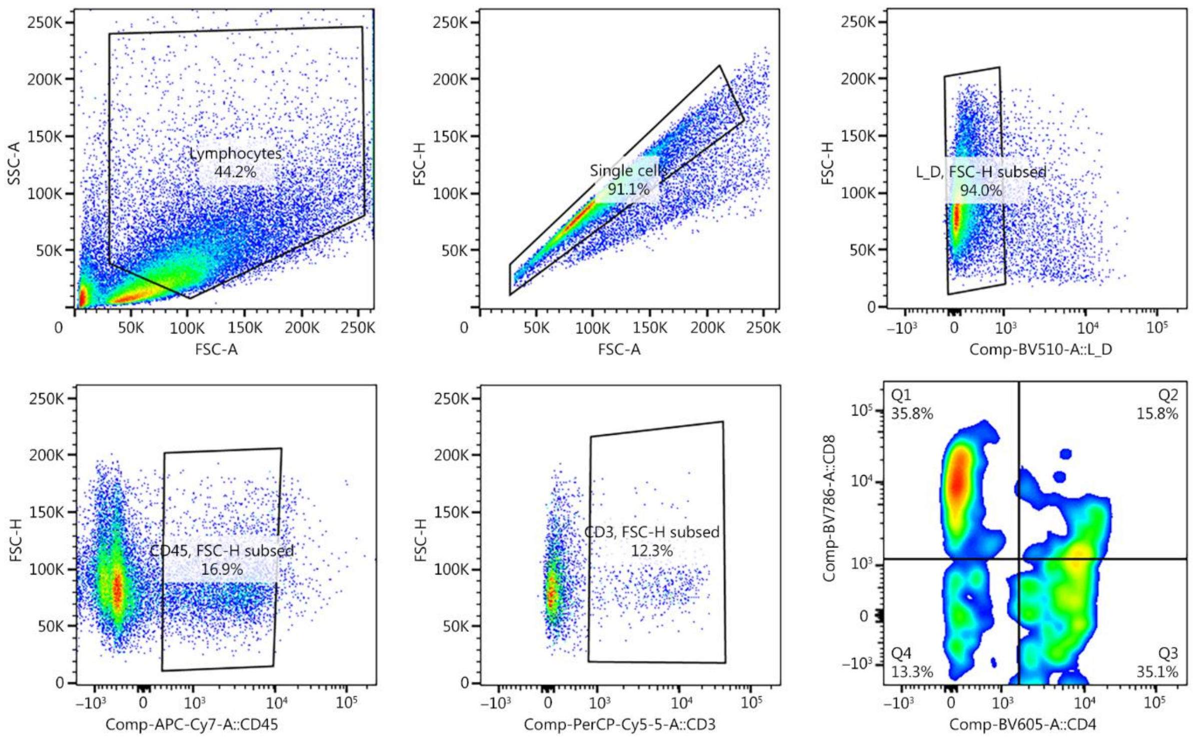

Fig. S33 Representative gating strategy of spleen lymphocytes for CD8^{+} T cell assay

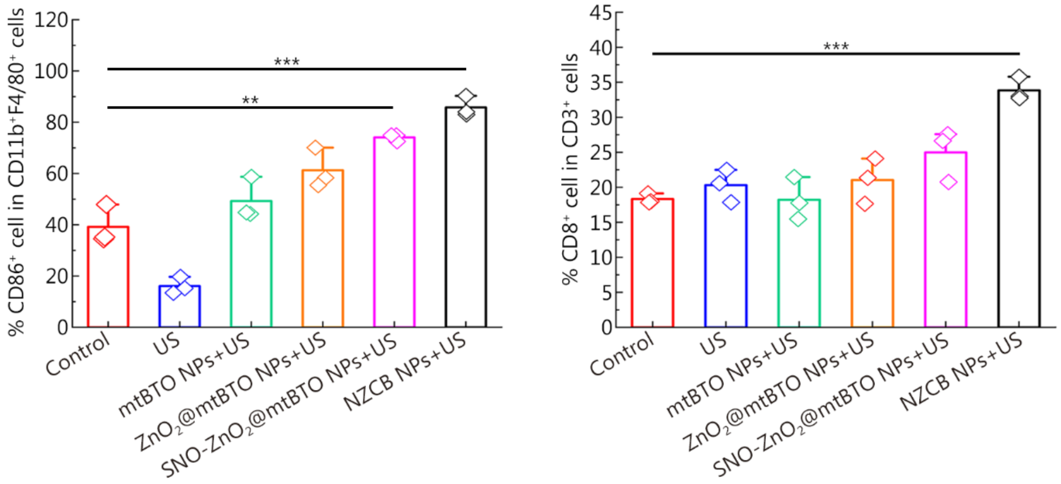

Fig. S34 Statistical analysis of CD86$^+$ cells in CD11b + F4/80$^+$ cells and CD8$^+$ T cells in CD3$^+$ T cells isolated from spleen tissues of mice after treatments of saline, US, mtBTO NPs + US, ZnO_2@mtBTO NPs + US, SNO-ZnO_2@mtBTO NPs + US, and NZCB NPs + US. $^{**}P < 0.01$, $^{***}P < 0.001$. NZCB S-nitrosothiols (SNO)-zinc peroxide (ZnO_2)@cyclic dinucleotide (CDN)@mesoporous tetragonal barium titanate (mtBTO), US ultrasound, NPs nanoparticles

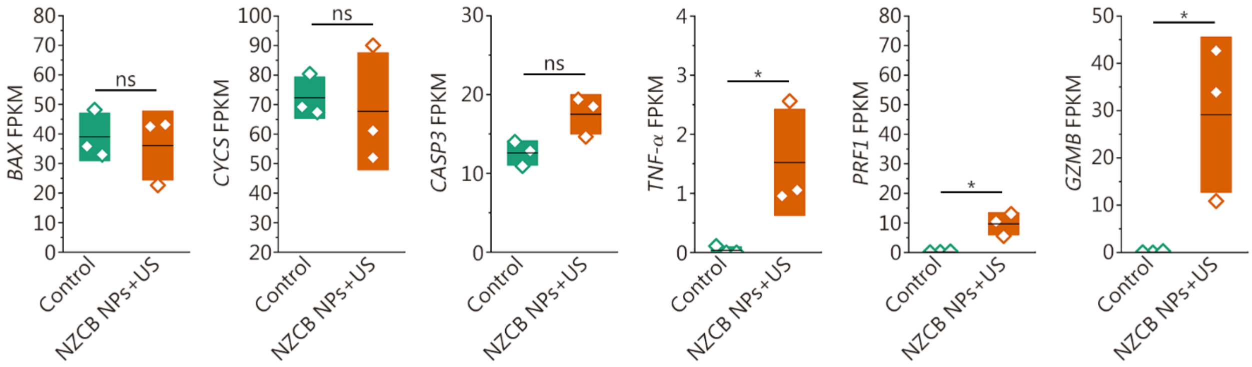

Fig. S35 Expression of apoptosis-associated genes, including *BAX*, *CYCS*, *CASP3*, *TNF-α*, *PRF1*, and *GZMB* between the control group and NZCB NPs + US group. $^{*}P < 0.05$, ns non-significant. BAX Bcl-2-associated X protein, CYCS cytochrome c, CASP3 caspase-3, TNF-α tumor necrosis factor-α, PRF1 perforin-1, GZMB granzyme B, NZCB S-nitrosothiols (SNO)-zinc peroxide (ZnO_2)@cyclic dinucleotide (CDN)@mesoporous tetragonal barium titanate (mtBTO), US ultrasound, NPs nanoparticles

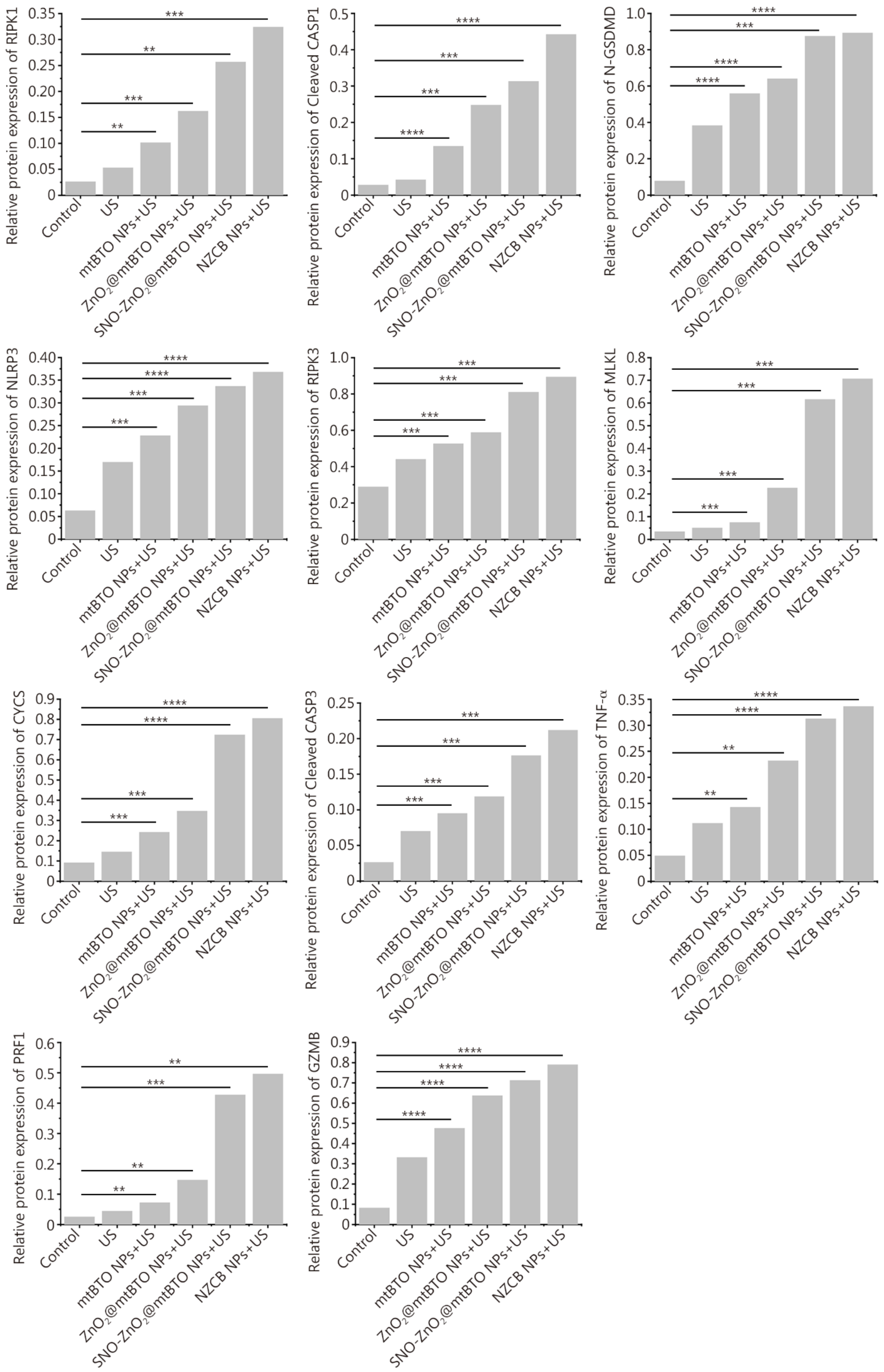
Relative protein expression of RIPK1
0.35
0.30
0.25
0.20
0.15
0.10
0.05
0
***
**
***
**
Relative protein expression of Cleaved CASP1
0.5
0.4
0.3
0.2
0.1
0
****
***
***
****
Relative protein expression of N-GSDMD
1.0
0.8
0.6
0.4
0.2
0
****
***
****
****
Relative protein expression of NLRP3
0.40
0.35
0.30
0.25
0.20
0.15
0.10
0.05
0
****
****
***
***
Relative protein expression of RIPK3
1.0
0.8
0.6
0.4
0.2
0
***
***
***
***
Relative protein expression of MLKL
0.8
0.7
0.6
0.5
0.4
0.3
0.2
0.1
0
***
***
***
***
Relative protein expression of CYCS
0.9
0.8
0.7
0.6
0.5
0.4
0.3
0.2
0.1
0
****
****
***
***
Relative protein expression of Cleaved CASP3
0.25
0.20
0.15
0.10
0.05
0
***
***
***
***
Relative protein expression of TNF-α
0.35
0.30
0.25
0.20
0.15
0.10
0.05
0
****
****
**
**
Relative protein expression of PRF1
0.6
0.5
0.4
0.3
0.2
0.1
0
**
***
**
**
Relative protein expression of GZMB
0.9
0.8
0.7
0.6
0.5
0.4
0.3
0.2
0.1
0
****
****
****
****
Control
US
mtBTO NPs+US
ZnO2@mtBTO NPs+US
SNO-ZnO2@mtBTO NPs+US
NZCB NPs+US

Fig. S36 Relative protein expression of PANoptosis-related proteins including RIPK1, CASP1, N-GSDMD, NLRP3, RIPK3, MLKL, CYCS, CASP3, TNF-α, PRF1 and GZMB after treatments of saline, US, mtBTO NPs + US, ZnO_2@mtBTO NPs + US, SNO-ZnO_2@mtBTO NPs + US, and NZCB NPs + US. $^{**}P < 0.01$, $^{***}P < 0.001$, $^{****}P < 0.0001$. RIPK1 receptor-interacting serine/threonine-protein kinase 1, CASP1 caspase-1, N-GSDMD N-terminal gasdermin D, NLRP3 NLR family pyrin domain containing 3, RIPK3 receptor-interacting serine/threonine-protein kinase 3, MLKL mixed lineage kinase domain-like protein, CYCS cytochrome c, TNF-α Tumor necrosis factor-α, PRF1 perforin-1, GZMB granzyme B, NZCB S-nitrosothiols (SNO)-zinc peroxide (ZnO_2)@cyclic dinucleotide (CDN)@mesoporous tetragonal barium titanate (mtBTO), US ultrasound, NPs nanoparticles

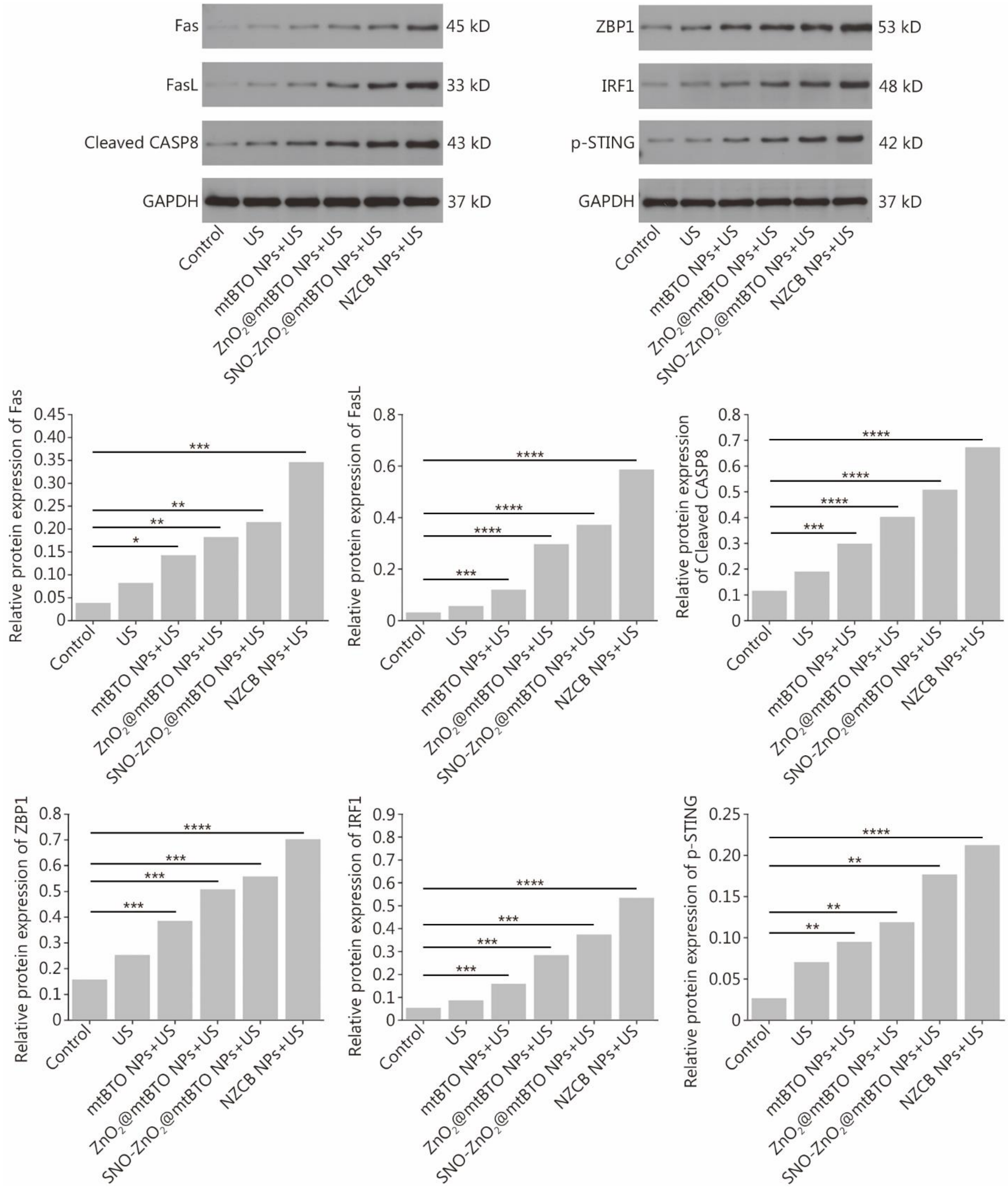

Fig. S37 Western blotting and quantitative analysis of proteins of Fas, FasL, cleaved CASP8, ZBP1, IRF1, and p-STING in different groups. $^{*}P < 0.05$, $^{**}P < 0.01$, $^{***}P < 0.001$, $^{****}P < 0.0001$. Fas Fas cell surface death receptor, FasL Fas ligand, cleaved CASP8 cleaved caspase-8, ZBP1 Z-DNA binding protein 1, IRF1 interferon regulatory factor 1, p-STING phosphorylated Stimulator of interferon genes, NZCB S-nitrosothiols (SNO)-zinc peroxide (ZnO_2)@cyclic dinucleotide (CDN)@mesoporous tetragonal barium titanate (mtBTO), US ultrasound, NPs nanoparticles

Table S1 Primers used for RT-PCR analysis

Target	Direction	Primer sequence (5' – 3')
BAX	Forward	AGACAGGGGCCTTTTTGCTAC
	Reverse	AATTCGCCGGAGACACTCG
RIPK1	Forward	GAAGACAGACCTAGACAGCGG
	Reverse	CCAGTAGCTTCACCACTCGAC
NLRP3	Forward	ATGCTGCTTCGACATCTCCT
	Reverse	AACCAATGCGAGATCCTGAC
GSDMD	Forward	CCATCGGCCTTTGAGAAAGTG
	Reverse	ACACATGAATAACGGGGTTTCC
TNF-α	Forward	CCCTCACACTCAGATCATCTTCT
	Reverse	GCTACGACGTGGGCTACAG
Actin	Forward	AGGTCGGTGTGAACGGATTTG
	Reverse	TGTAGACCATGTAGTTGAGGTCA

BAX BCL2-associated X protein, *RIPK1* receptor-interacting serine/threonine-protein kinase 1, *NLRP3* NLR family pyrin domain containing 3, *GSDMD* gasdermin D, *TNF-α* tumor necrosis factor-α
